# The efficiency of universal mitochondrial DNA barcodes for species discrimination of *Pomacea canaliculata* and *Pomacea maculata*

**DOI:** 10.7717/peerj.8755

**Published:** 2020-04-01

**Authors:** Adrian Kannan, Suganiya Rama Rao, Shyamala Ratnayeke, Yoon-Yen Yow

**Affiliations:** Department of Biological Sciences, School of Science & Technology, Sunway University, Selangor Darul Ehsan, Malaysia

**Keywords:** Invasive apple snails, *Pomacea*, DNA barcoding, Mitochondrial markers, COI, 16S rDNA

## Abstract

Invasive apple snails, *Pomacea canaliculata* and *P. maculata*, have a widespread distribution globally and are regarded as devastating pests of agricultural wetlands. The two species are morphologically similar, which hinders species identification via morphological approaches and species-specific management efforts. Advances in molecular genetics may contribute effective diagnostic tools to potentially resolve morphological ambiguity. DNA barcoding has revolutionized the field of taxonomy by providing an alternative, simple approach for species discrimination, where short sections of DNA, the cytochrome c oxidase subunit I (COI) gene in particular, are used as ‘barcodes’ to delineate species boundaries. In our study, we aimed to assess the effectiveness of two mitochondrial markers, the COI and 16S ribosomal deoxyribonucleic acid (16S rDNA) markers for DNA barcoding of *P. canaliculata* and *P. maculata*. The COI and 16S rDNA sequences of 40 *Pomacea* specimens collected from six localities in Peninsular Malaysia were analyzed to assess their barcoding performance using phylogenetic methods and distance-based assessments. The results confirmed both markers were suitable for barcoding *P. canaliculata* and *P. maculata*. The phylogenies of the COI and 16S rDNA markers demonstrated species-specific monophyly and were largely congruent with the exception of one individual. The COI marker exhibited a larger barcoding gap (6.06–6.58%) than the 16S rDNA marker (1.54%); however, the magnitude of barcoding gap generated within the barcoding region of the 16S rDNA marker (12-fold) was bigger than the COI counterpart (approximately 9-fold). Both markers were generally successful in identifying *P. canaliculata* and *P. maculata* in the similarity-based DNA identifications. The COI + 16S rDNA concatenated dataset successfully recovered monophylies of *P. canaliculata* and *P. maculata* but concatenation did not improve individual datasets in distance-based analyses. Overall, although both markers were successful for the identification of apple snails, the COI molecular marker is a better barcoding marker and could be utilized in various population genetic studies of *P. canaliculata* and *P. maculata*.

## Introduction

Cryptic species are often subject to ambiguous and erroneous species classification in the field of biological taxonomy. Traditional taxonomical hypotheses have relied solely on the use of morphological features as evolutionary evidence of speciation events and subsequently, as the defining criteria for species delimitation (Hebert & Gregory, 2005). In the case of cryptic speciation, the identification of genetically distinct species is masked empirically by their indistinguishable morphology which renders traditional taxonomy sometimes impractical (Bickford et al., 2007). The increasing significance of molecular genetics in modern taxonomy has uncovered hidden or cryptic biodiversity to aid and improve the resolution of morphological-based species delimitation (Bickford et al., 2007), particularly since the introduction of DNA barcoding (Hebert et al., 2003). The integration of short stretches of DNA termed ‘barcodes’ in delineating species boundaries has enabled accurate and rapid identification of various species (Vences et al., 2005; Schäffer, Zachos & Koblmüller, 2017; Tizard et al., 2019). Although prone to pitfalls (Will, Mishler & Wheeler, 2005; Waugh, 2007), DNA barcoding is sometimes considered a valuable tool because it complements traditional morphological taxonomy facilitating an integrated approach of species delimitation (Sheth & Thaker, 2017). The role of DNA barcoding as a diagnostic tool for cryptic diversity in conservation biology is particularly useful, since it plays a crucial role in managing invasive alien pests (Armstrong & Ball, 2005).

*Pomacea canaliculata* and *P. maculata,* family Ampullariidae (apple snails), are two pantropical species of invasive freshwater gastropods that are best known for their damage to wetland agriculture. Originally from South America, anthropogenic dispersal via the aquarium trade and as a protein source for humans have enabled these species to breach geographical barriers and establish successful populations in many parts of the world including several states in the United States as well as some Asian and European countries (De Brito & Joshi, 2016). In Malaysia, *P. canaliculata* and *P. maculata* are serious pests of rice agriculture (Salleh et al., 2012; Arfan et al., 2014; Arfan et al., 2016), causing losses of approximately RM 82 (US$ 20) million (2010) in rice fields in the peninsula (Yahaya et al., 2017). In addition, *P. canaliculata* and to a lesser extent, *P. maculata* pose health concerns: the snails are intermediate hosts of the rat lung worm *Angiostrongylus cantonensis*, which can infect humans (Lv et al., 2009; Yang, Wu & Lun, 2013; Teem et al., 2013; Hu et al., 2018). Also, both species of snails are capable of rapidly depleting freshwater macrophytes and therefore capable of disrupting the integrity and ecosystem function of freshwater wetlands (Carlsson, Brönmark & Hansson, 2004; Kwong, Chan & Qiu, 2009).

Effective diagnostic tools for species identification of these invasive snails are important to aid detection and control efforts. Several species-specific quantitative and qualitative phenotypic characters of *P. canaliculata* and *P. maculata* were identified by Hayes et al. (2012). Rama Rao et al. (2018) confirmed that specimens of *P. canaliculata* and *P. maculata* in Peninsular Malaysia could not be reliably assigned to species based on the proposed phenotypic characteristics. In Malaysia, studies to identify species of *Pomacea* relied mostly on conventional morphological approaches (Salleh et al., 2012; Arfan et al., 2014; Arfan et al., 2016; Yahaya et al., 2017); thus, these two species were probably misidentified as in other regions (Horgan, Stuart & Kudavidanage, 2014). High interspecific similarity and intraspecific variation in both species have impeded morphological delineation of species (Estebenet & Martin, 2003; Cowie, Hayes & Thiengo, 2006; Rawlings et al., 2007) which may be influenced by different biological and environmental factors in both native and invaded ranges (Valentin et al., 2018).

DNA barcoding has been the diagnostic tool of choice to assess variation in the genetic composition of organisms for species delineation. Generally, mitochondrial DNA is a reliable marker for evolutionary genetics due to its rapid evolutionary rate, high copy number and lack of genetic recombination (Brown, George & Allan, 1979; Simon et al., 1994; Arif et al., 2011). Additionally, the putative maternal and haploid mode of mitochondrial inheritance provides a single evolutionary lineage thereby reducing complexities of genetic recombination which can complicate phylogenetic reconstruction for diploid loci (Ladoukakis & Zouros, 2017). A portion of the cytochrome c oxidase subunit I (COI) gene, the universal DNA barcoding marker (Hebert et al., 2004; Savolainen et al., 2005; Ratnasingham & Hebert, 2007), has successfully delineated *P. canaliculata* from *P. maculata* from native (Brazil and Argentina) and invaded (China, Japan, USA and Malaysia) regions (Hayes, Cowie & Thiengo, 2009; Matsukura et al., 2008; Rama Rao et al., 2018). Furthermore, the COI barcoding region provided successful species delineation and it was concordant with morphological-based assessments (Hayes et al., 2012), which serves as a prerequisite for the reliable use of DNA barcodes. The COI region has also been utilised in genetic diversity studies (Hayes et al., 2008; Yang et al., 2018) as well as in rapid identification approaches (Matsukura et al., 2008; Cooke et al., 2012).

The 16S ribosomal deoxyribonucleic acid (rDNA) is a well-known barcoding marker for inferring phylogenetic relationships among bacterial species (Weisburg et al., 1991; O’Neill et al., 1992; Janda & Abbott, 2007) and is increasing in its application among animal species (Vences et al., 2005; Lim et al., 2012; Xia et al., 2011; Wang et al., 2018). In fact, 16S rDNA has been utilized for species delineation in the family Ampullariidae (Thaewnon-Ngiw et al., 2004; Jørgensen, Kristensen & Madsen, 2008; Hayes et al., 2012; Li, Bian & Zhao, 2013). However, in Peninsular Malaysia, the only barcoding marker employed for *Pomacea* spp. is the COI barcoding region (Hayes et al., 2008; Rama Rao et al., 2018) and this limits comparison with other barcoding markers such as the 16S rDNA to determine its usefulness. In addition, the sole use of the COI barcoding marker for species identification may result in inaccurate identification if properties of the barcoding region do not sufficiently resolve intra- versus interspecific sequence variability (Vences et al., 2005). Thus, an additional marker should be employed, such as the 16S rDNA marker, which could potentially complement the COI molecular interpretation for better evaluation of species identification.

In the present study, we evaluated the effectiveness of both mitochondrial markers of COI and 16S rDNA for DNA barcoding of *Pomacea* spp. in Peninsular Malaysia. We then used sequence data from both markers to assess phylogenetic relationships of *P. canaliculata* and *P. maculata* from Peninsular Malaysia, including conspecifics and congeners from native and invaded regions

## Materials & Methods

### Sample collection and processing

**Figure 1:**
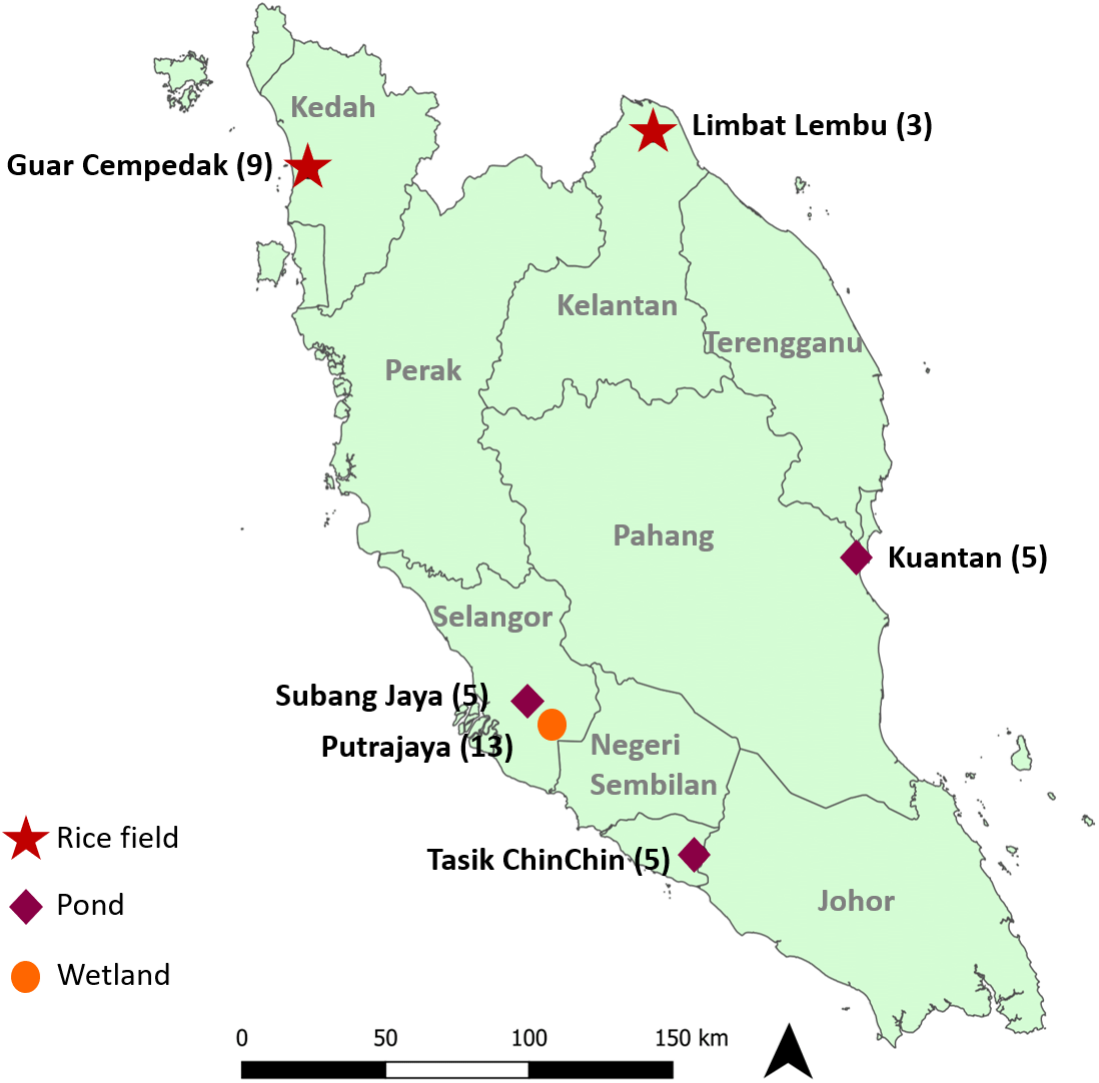
Geographical location of study sites in Peninsular Malaysia where specimens were collected. Numbers next to the locations indicate the number of specimens analysed in this study and symbols represent the type of location.

Experimental design and procedures of the study were approved by the Sunway University Research Ethics Committee (Approval code: PGSUREC 2018/044). Three to 13 *Pomacea* spp. were collected from six geographical locations (Fig. 1 & Table 1) in Peninsular Malaysia from February 2016 to September 2019. Sample collection in Taman Wetlands Putrajaya was granted by the Environmental, Lake and Wetland Division (Approval reference number: PPj/R/A/TWH/69(14)). No specific permission was required for the other locations as it did not involve any protected species. Approximately 1 to 5 mg of foot tissue was immersed in distilled water for about two hours to soften the fibres, then chopped and homogenized.

**Table 1:** List of *Pomacea* species, sampling locations and GenBank accession number.

**Sequence ID/ Species**	**Location**	**GenBank accession number**	
		**COI**	**16S rDNA**
AN1	Kuantan, Pahang (*n* = 5)	MN623417	MN623441
AN2		MN623418	MN623442
AN3		MN623419	MN623443
AN4		MN623420	MN623444
AN5		MN623421	MN623445
CC1	Tasik ChinChin, Melaka (*n* = 5)	MN623422	MN623446
CC2		MN623423	MN623447
CC3		MN623424	MN623448
CC4		MN623425	MN623449
CC5		MN623426	MN623450
SJ2	Subang Jaya, Selangor (*n* = 5)	MG230743	MN623451
SJ5		MG230744	MN623452
SJ7		MG230745	MN623453
SJ13		MG230746	MN623454
SJ14		MG230747	MN623455
LL2	Limbat Lembu, Kelantan (*n* = 3)	MN623427	MN623456
LL5		MN623428	MN623457
LL6		MN623429	MN623458
PJ1	Putrajaya, Selangor (*n* = 13)	MG230763	MN623468
PJ2		MG230764	MN623469
PJ3		MG230765	MN623470
PJ4		MG230766	MN623471
PJ5		MG230767	MN623472
PJ11		MG230771	MN623473
PJ13		MN623435	MN623475
PJ14		MN623436	MN623474
PJ15		MG230773	MN623476
PJ16		MN623437	MN623477
PJ20		MN623438	MN623478
PJ29		MN623439	MN623479
PJ45		MN623440	MN623480
GC1	Guar Cempedak, Kedah (*n* = 9)	MG230788	MN623459
GC2		MG230789	MN623460
GC5		MG230791	MN623461
GC6		MN623430	MN623462
GC7		MG230792	MN623463
GC8		MN623431	MN623464
GC11		MN623432	MN623465
GC18		MN623433	MN623466
GC20		MN623434	MN623467
*P. canaliculata* *	Malaysia	MG230755	–
	Singapore	KY081757	–
	Indonesia	KY574007	–
	China	FJ946820	–
		FJ946823	–
		–	KJ766112
		–	KF002499
	Hong Kong	KT313034	–
	Japan	AB433773	–
	Philippines	–	EU274501
		EU528483	–
	Chile	KX965671	–
	USA	EF514982	
	Argentina	AB728574	–
		FJ710314	–
		FJ710315	–
		EU528529	–
		–	FJ710235
	Uruguay	FJ710313	–
*P. maculata* *	Malaysia	MG230787	–
	Singapore		KY081737
	China	FJ946828	–
	Japan	AB433781	–
	Spain	GU236491	–
	USA	JX845573	–
	Brazil	EU528568	FJ710229
		–	FJ710228
*P. lineata* *	Brazil	FJ710309	FJ710231
		FJ710310	FJ710230
		FJ710311	–
*P. paludosa* *	USA	EF514960	–
		EU528590	FJ710237
		EU528591	FJ710238
*P. scalaris* *	Argentina	EU528506	FJ710240
	Brazil	FJ710316	FJ710241

**Notes.**

* GenBank sequences retrieved from the NCBI database that were used as reference sequences in the phylogenetic analyses and similarity DNA identification techniques.

- Sequences that were not used in the analyses or unavailable.

### Genomic DNA extraction, amplification and sequencing

Genomic DNA was extracted from the foot tissue using NucleoSpin^®^Tissue kit (Marcherey-Nagel, Germany) according to manufacturer’s instruction. The concentration and purity of the extracted genomic DNA was measured using Biodrop µlite (United Kingdom). The eluted genomic DNA for each specimen was stored at −20 °C for future use. The isolated genomic DNAs were used as templates in PCR amplification for both COI and 16S rDNA markers. A 700 bp fragment of the COI barcoding region was amplified using forward LCO1490 (5′-GGTCAACAAATCATAAAGATATTG-3′) and reverse HCO2198 primers (5′TAAACTTCAGGGTGACCAAAAAATCA-3′) (Folmer et al., 1994). A 600 bp fragment of the 16SrDNA barcoding region was amplified using forward 16F (5′- CGCCTGTTTATCAAAAACAT-3′) and reverse 16R primers (5′- CCGGTCTGAACTCAGATCACGT-3′) (Palumbi et al., 1991). DNA amplifications were performed in a final volume of 25 µL containing 12.5 µL Prime Taq Premix (2X) (GENETBIO Inc, Korea), 1 µL of 10 pmol/µL forward and reverse primers, 1 µL extracted DNA sample and 9.5 µL distilled water. The PCR parameters used were as described in Folmer et al. (1994) and Palumbi et al. (1991). Amplification were carried out using T100^®^ Thermal Cycler (Bio-Rad Laboratories.Inc, USA). All amplified PCR products were electrophoresed on 1% agarose gel to quantify the band sizes. PCR products with the expected band sizes were sent to MyTACG Bioscience Enterprise for sequencing with the COI (Folmer et al., 1994) and 16S rDNA primers (Palumbi et al., 1991).

### Data analyses

#### Phylogenetic analysis

Phylogenetic relationships of the COI and 16S rDNA sequences were analysed for species identification. Species were successfully assigned if the reference sequences formed clusters of conspecific sequences that constitutes a monophyletic clade. Forward and reverse DNA sequencing chromatograms were examined, edited and assembled into contiguous sequences using ChromasPro2.0 (Technelysium Pty.Ltd., Australia) and BioEdit 7.0.5.3 (Hall, 1999). COI data from Phoong et al. (2018) and Rama Rao et al. (2018) for 12 and four specimens respectively, were employed in this study with the accession numbers (MG230743–MG230792) in Table 1. To reconstruct the COI and 16S rDNA phylogeny in this study, 29 COI and 13 16S rDNA GenBank reference sequences of *Pomacea* spp. were retrieved from the National Center for Biotechnology Information (NCBI) database (Table 1). The COI GenBank reference sequences were cross-referenced with the Barcoding of Life Data System (BOLD) database (Table S1). The assembled consensus specimen sequences along with the GenBank sequences were aligned using ClustalX (Larkin et al., 2007) to generate COI and 16S rDNA datasets. Additionally, we combined the COI and 16S rDNA sequences to produce an aligned dataset of concatenated mitochondrial barcoding markers to evaluate whether barcoding performance improved over single marker counterparts. The COI, 16S rDNA and COI + 16S rDNA datasets were subject to ML tree reconstruction via PhyML 3.0 (Guindon et al., 2010) by implementing the Smart Model Selection (SMS) (Lefort, Longueville & Gascuel, 2017) using the Akaike Information Criterion (AIC) with 1000 bootstrap replicates. Phylogenetic trees were also reconstructed based on Bayesian inference (BI) to confirm the topology inferred by the ML approach. Kakusan version 3.0 (Tanabe, 2007) was employed to output the best fit DNA substitution models using the Bayesian Information Criterion (BIC). Two independent runs using four Markov Chain Monte Carlo (MCMC) for 10,000,000 generations were run in MrBayes v3.2.1 (Huelsenbeck et al., 2001) with convergence diagnostics calculated every 5000th generation. Trees in each chain were sampled every 500th generation. Topologies were discarded as burn-in after evaluating the convergence in Tracer version 1.5.0 (Rambaut & Drummond, 2009). The reconstructed phylogenetic trees were viewed using FigTree v.1.4.0 (Rambaut, 2012). *P. scalaris* (EU528506, FJ710240, FJ710316 and FJ710241) was used as an outgroup to root the phylogenies.

#### Pairwise genetic distance analysis

COI, 16S rDNA and COI + 16S rDNA sequences of *Pomacea* spp. were aligned by species according to the species delineation provided by the phylogenetic analyses. The uncorrected pairwise distance and Kimura-2-parameter (K2P) intra- and interspecific distances were calculated for the aligned datasets via MEGA7 (Molecular Evolutionary Genetics Analysis Version 7.0) program (Kumar, Stecher & Tamura, 2011) and TaxonDNA Species Identifier v1.7.7 (Meier et al., 2006). Barcoding gaps or the differences between inter- and intraspecific sequence variation, were assessed in two ways. In the first, species boundaries were effectively delineated when the lowest interspecific distance exceeded the highest intraspecific distance. Analyses of all sequences with at least 300 bp overlap were carried out where pairwise distances (i) included all intraspecific and interspecific distances and (ii) excluded 5% of the largest intraspecific and 5% smallest interspecific distances (90% pairwise genetic distances) (Meier et al., 2006; Meier, Zhang & Ali, 2008). The second, the magnitude of the barcoding gap, if any, was assessed when the mean interspecific distance exceeded the mean intraspecific distance by 10 folds (Hebert et al., 2004).

#### Sequence similarity analysis

Three similarity-based DNA distance approaches, ‘best match’, ‘best close match’ and ‘all species barcode’ (Meier et al., 2006) were conducted in TaxonDNA Species Identifier v1.7.7 to further evaluate the performance of the individual COI and 16S rDNA barcoding markers based on sequence similarity. Reference sequences or ‘barcodes’ of *P. canaliculata*, *P. maculata*, *P. lineata*, *P. paludosa* and *P. scalaris* retrieved from GenBank (Table 1) were used to enable comparison with the query sequences. A threshold value where 95% of all intraspecific distances were found, was set for both ‘best close match’ and ‘all species barcode’ analyses, where species delineation is successful if the barcode match is below the threshold. The analyses were not conducted on the COI + 16S rDNA dataset because reference sequence data for concatenated COI and 16S rDNA markers from the same individual were scarce.

## Results

The COI and 16S rDNA barcoding regions from 40 specimens were successfully amplified and sequenced. Sizes of amplified fragments of these regions were in the range of 669 bp to 714 bp and 528 bp to 550 bp for the COI and 16S rDNA regions respectively. The aligned sequences with 612 bp for COI and 472 bp for 16S rDNA datasets were used in all subsequent analyses. The aligned sequences in the concatenated dataset of 1084 bp consisted of 612 bp and 472 bp from the COI and 16S rDNA barcoding regions respectively and were used in the phylogenetic and barcoding gap analyses.

### Phylogenetic analyses

The aligned COI sequence reads of 40 *Pomacea* specimens in this study and 29 COI *Pomacea* spp. GenBank sequences produced a dataset with 158 sites phylogenetically informative sites.

**Figure 2:**
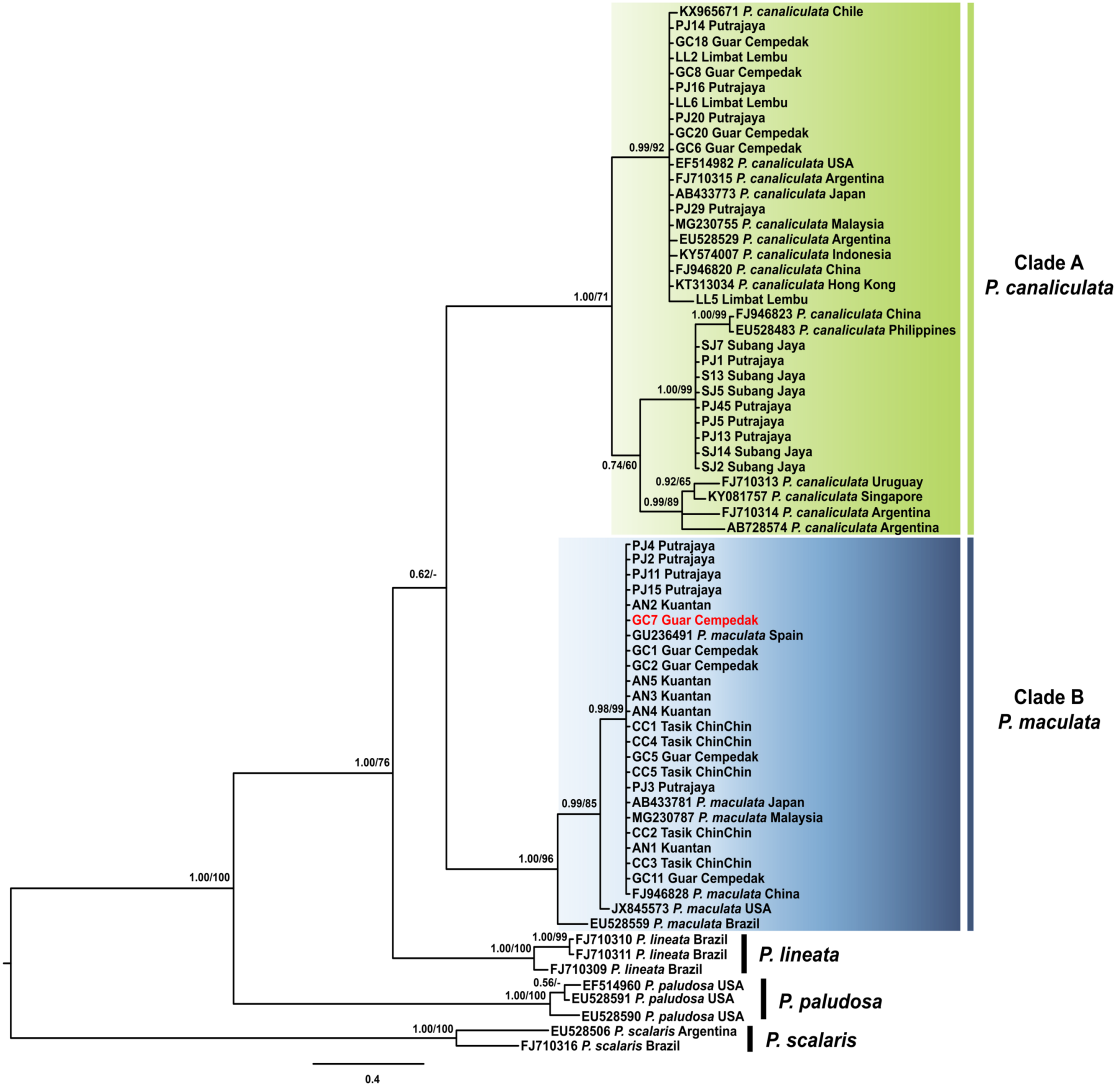
Phylogram shows Bayesian inference analysis of *P. canaliculata and P. maculata* from Peninsular Malaysia and *Pomacea* spp*.* from other regions based on COI marker. Kuantan, Tasik ChinChin, Limbat Lembu, Subang Jaya, Putrajaya and Guar Cempedak refer to geographic locations in Peninsular Malaysia where specimens in this study were collected. Numeric values at nodes are arranged in order of Bayesian posterior probabilities/ ML bootstrap support and ‘-’ indicates no values for ML bootstrap support.*****P. scalaris* was chosen as an outgroup taxon. Clades highlighted in green and blue are *P. canaliculata* and *P. maculata* clades respectively. The taxon in red indicates individual with COI-16S rDNA incongruent identification.

The best fit model for the ML-derived COI phylogenetic dataset was the generalized time reversible model including invariable sites (GTR + I) with a log likelihood value of −2402.13 for the preferred tree. For the BI analysis, the first 25% topologies were discarded as burn-in after the convergence was evaluated. The best fit model was the Hasegawa, Kishino and Yano model including invariable sites and a gamma distributed parameter (HKY + I + G) with a log likelihood value of −2492.62. The COI phylogenetic trees inferred via ML and BI had concordant topologies and resolved the ingroup sequences into four monophyletic clades: *P. paludosa, P. lineata*, *P. canaliculata* and *P. maculata* (Fig. 2). Both statistical analyses inferred *P. canaliculata* and *P. maculata* as sister taxa; however, the posterior probability in the BI approach was considerably low, whereas the bootstrap support in the ML analyses was insufficient to support the inferred relationship (below 50%). Sequences of specimens of this study were found in two of these clades; Clade A and Clade B with strong supports (Fig. 2). Twenty specimens from our study clustered in Clade A, which also contained *P. canaliculata* sequences from native (Argentina and Uruguay) and invaded (Malaysia, Singapore, Indonesia, Hong Kong, China, Japan, Chile and USA) regions. Deeper nodes indicated three genetically divergent clades of *P. canaliculata* where the specimens in our study were found in two. The first consisted of nine specimens from Peninsular Malaysia along with *P. canaliculata* individuals from China and Philippines. All other specimens were found in the other minor clade. In clade B, 20 specimens from our samples were homologous to *P. maculata* from native (Brazil) and invaded (Malaysia, China, Japan, Spain and USA) regions.

**Figure 3:**
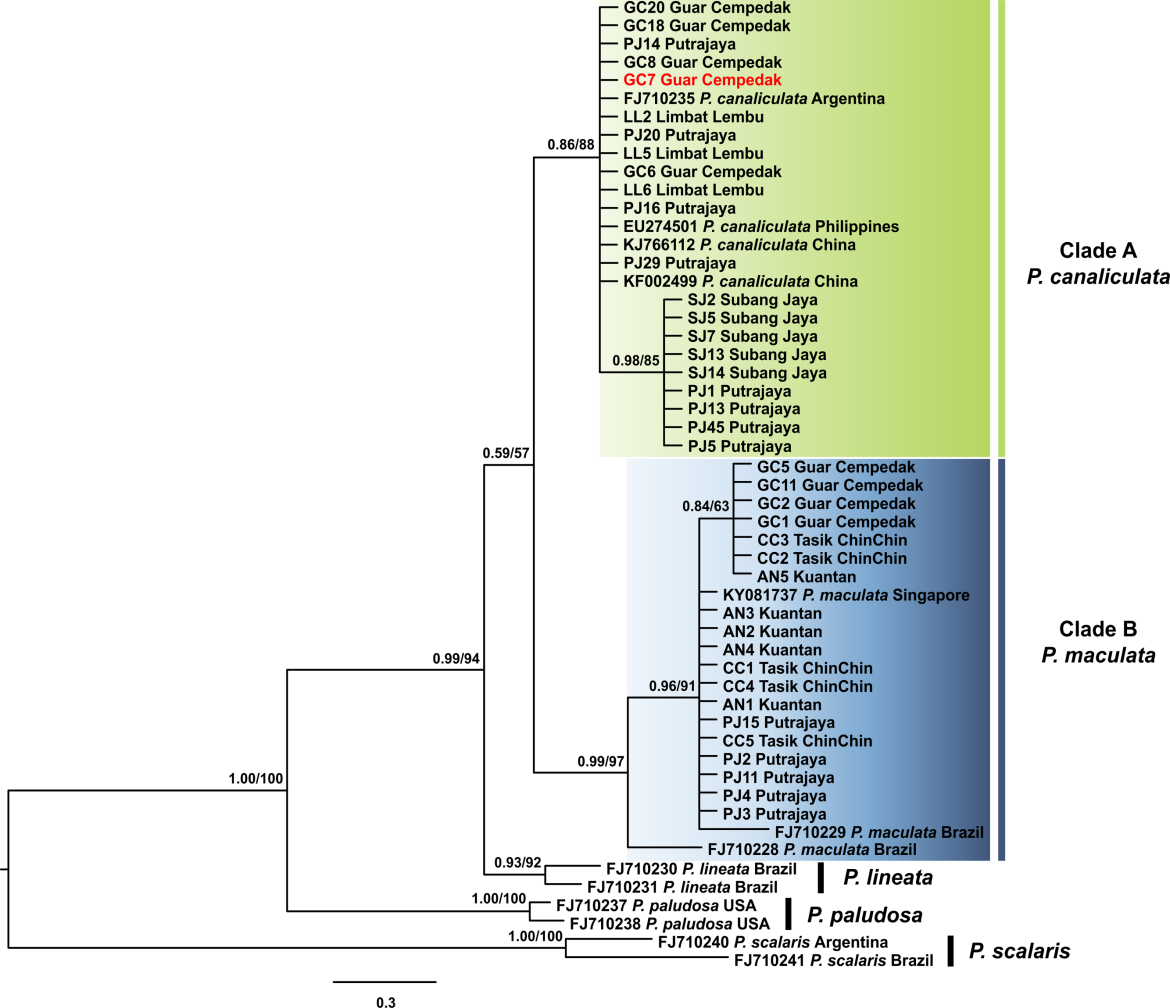
Phylogram shows Bayesian inference analysis of *P. canaliculata and P. maculata* from Peninsular Malaysia and *Pomacea* spp*.* from other regions based on 16S rDNA marker. Kuantan, Tasik ChinChin, Limbat Lembu, Subang Jaya, Putrajaya and Guar Cempedak refer to geographic locations in Peninsular Malaysia where specimens in this study were collected. Numeric values at nodes are arranged in order of Bayesian posterior probabilities/ ML bootstrap support.*****P. scalaris* was chosen as an outgroup taxon. Clades highlighted in green and blue are *P. canaliculata* and *P. maculata* clades respectively. The taxon in red indicates individual** with COI-16S rDNA incongruent identification.

The 16S rDNA sequence reads of 40 specimens in this study along with 13 *Pomacea* sequences from GenBank with 51 phylogenetically informative sites. The best fit model for the ML-derived 16S rDNA phylogenetic dataset was the GTR + I model and the log likelihood value of for the preferred tree was −1078.22. For the BI analysis, the first 25% topologies were discarded as burn-in after the convergence was evaluated. The best fit model was the HKY + G model with a log likelihood value of −1218.16. Concordant topologies were inferred from both BI and ML statistical approaches (Fig. 3). With *P. scalaris* rooted as the outgroup, the 16S rDNA phylogeny among the taxa suggested that species discrimination was successful where the GenBank sequences of *P. paludosa*, *P. lineata*, *P. canaliculata* and *P. maculata* clustered into their respective monophyletic clades. Sequences of specimens in our study fell into two well supported clades, Clade A and Clade B (Fig. 3). Twenty-one specimens were found in Clade A and clustered with published *P. canaliculata* GenBank sequences. Clade A was subsequently resolved into 2 clades in the ML tree, where the first clade consisted entirely of local specimens. The remaining *P. canaliculata* specimens from our study clustered with individuals from native (Argentina) and invaded (China and Philippines) regions; however, these specimens did not form a discrete clade using BI analysis (Fig. 3). The remaining 19 specimens clustered with GenBank sequences of *P. maculata* in Clade B with strong supports. Two native *P. maculata* sequences from Brazil suggest early divergence among native populations. Seven specimens from Peninsular Malaysia formed a small clade, whereas the remaining 12 specimens were unresolved along with *P. maculata* sequences from native (Brazil) and invaded (Singapore) regions. When both COI and 16S rDNA phylogenies were compared, one specimen (GC7) was identified as *P. maculata* based on the COI sequence, and as *P. canaliculata* based on its 16S rDNA sequence (Figs. 2 & 3).

**Figure 4:**
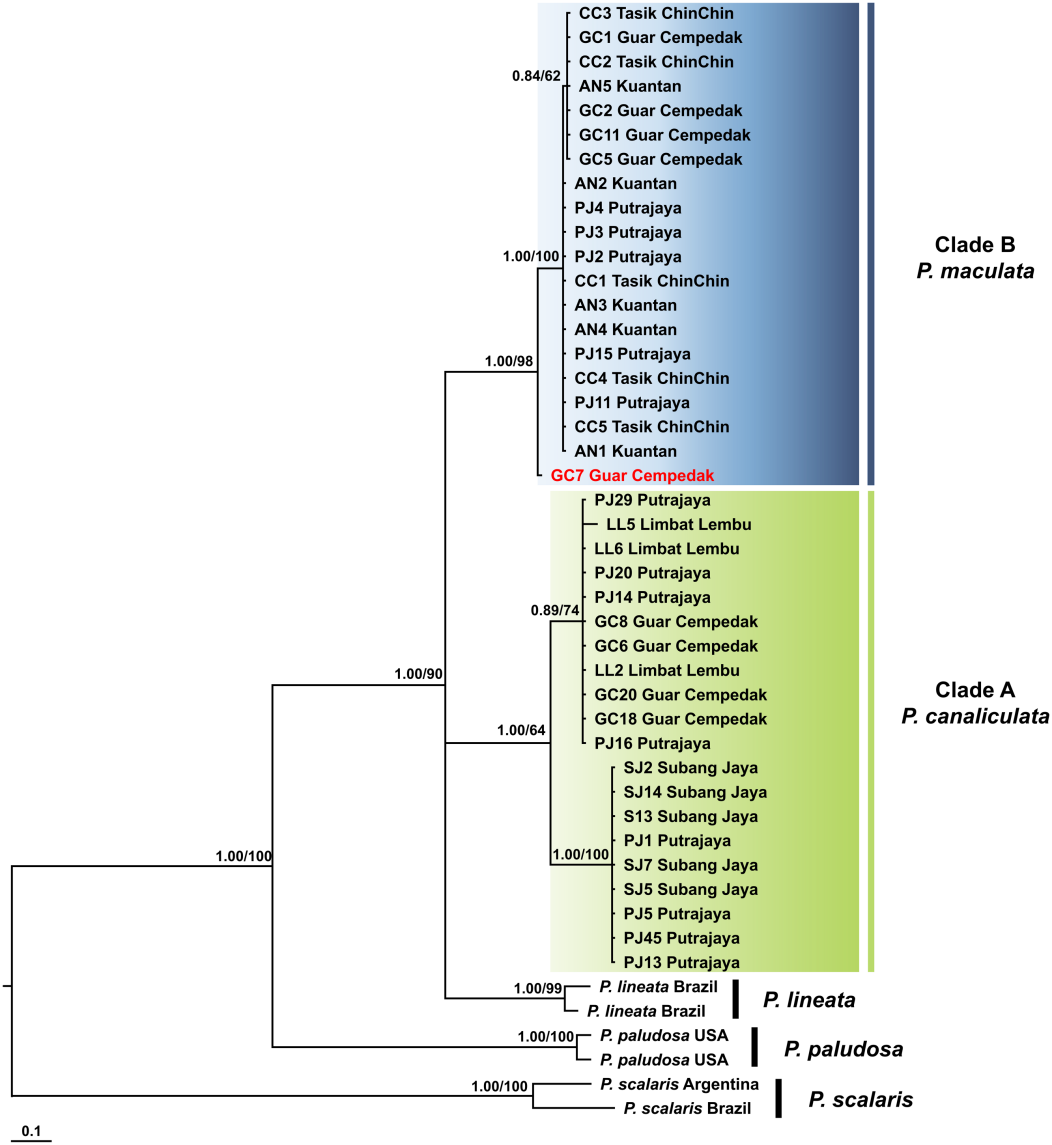
Phylogram shows Bayesian inference analysis of *P. canaliculata and P. maculata* from Peninsular Malaysia and *Pomacea* spp*.* from other regions based on the concatenated COI and 16S rDNA markers. Kuantan, Tasik ChinChin, Limbat Lembu, Subang Jaya, Putrajaya and Guar Cempedak refer to geographic locations in Peninsular Malaysia where specimens in this study were collected. Numeric values at nodes are arranged in order of Bayesian posterior probabilities/ ML bootstrap support.*****P. scalaris* was chosen as an outgroup taxon. Clades highlighted in green and blue are *P. canaliculata* and *P. maculata* clades, respectively. The taxon in red indicates individual** with COI-16S rDNA incongruent identification from the single marker analyses.

The COI + 16S rDNA dataset consisted of all 40 specimens and six concatenated sequences (COI and 16S rDNA) of *Pomacea* from GenBank. The concatenated dataset consisted of 200 phylogenetically informative sites. The best fit model for the ML-derived concatenated phylogenetic dataset was the GTR + I model and the log likelihood value for the preferred tree was −3145.33. For the BI analysis, the first 25% topologies were discarded as burn-in after the convergence was evaluated. The best fit models for each partition were used for the concatenated dataset and the log likelihood value of the preferred tree was −3238.71. Species discrimination via monophyly was successful with concordant topologies inferred from the ML and BI analyses (Fig. 4). The phylogeny inferred *P. canaliculata*, *P. maculata* and *P. lineata* as monophyletic multifurcation products of a common ancestor. The specimens in our study clustered in two strongly supported clades, Clade A and B, each with 20 specimens. When the single gene phylogenies of COI and 16S rDNA (Figs. 2 and 3) were used as references for species identification, specimens in Clade A and Clade B were inferred as *P. canaliculata* and *P. maculata* respectively. When the phylogeny-derived species identity of each specimen was compared, no discrepancies occurred in the COI and COI + 16S rDNA phylogenetic trees (Figs. 2 and 4).

### Barcoding region characteristics

The aligned COI dataset consisted of 612 bp with 77 variant characters of which 73 (11.9%) characters were parsimony informative. However, four singleton variable characters (0.7%) were detected from an individual sequence with no conspecific representation in the aligned dataset (Table 2). These bases were ambiguous as depicted by their respective peaks on the chromatogram. The 16S rDNA barcoding region (472 bp) consisted of 13 variant characters of which all (2.8%) were parsimony informative. The datasets showed that the COI had a higher rate of nucleotide variance compared to the 16S rDNA marker. Although the variation percentage was low for the 16S rDNA barcoding region, the variation was sufficient for species delineation. The COI + 16S rDNA dataset (1084 bp) from the concatenation of the COI (56.5%) and 16S rDNA (43.5%) barcoding regions consisted of 86 (7.9%) parsimony informative and 4 (0.4%) singleton variable characters. The concatenation of the barcoding markers resulted in a 5.1% increase in nucleotide variance rate from the relatively less variant 16S rDNA marker but a 4% decrease from the relatively variant COI marker.

### DNA barcoding gap assessment

**Figure 5:**
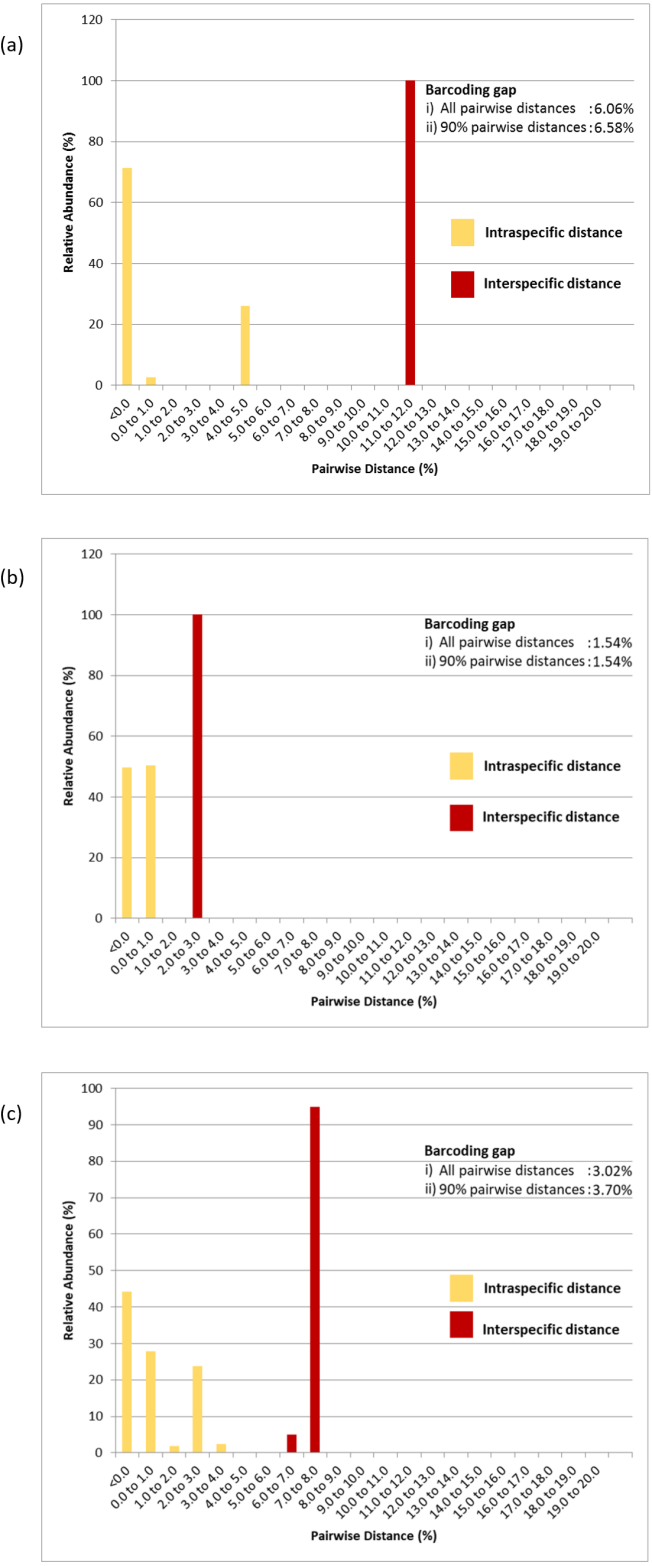
Relative abundance of intra- and interspecific K2P pairwise distances of (A) COI, (B) 16S rDNA and (C) COI + 16S rDNA aligned datasets. Numeric values represent the barcoding gaps generated when the lowest interspecific distance exceeded the highest intraspecific distance. Barcoding gaps were calculated with all pairwise genetic distances included and when the largest 5% intraspecific and the lowest 5% interspecific pairwise distances were excluded.

Pairwise distance distributions encompassing all intra- and interspecific distances of the COI (Fig. 5A), 16S rDNA (Fig. 5B) and COI + 16S rDNA (Fig. 5C) datasets were plotted. The interpretation of pairwise genetic distances between two specimens was based on the K2P model because uncorrected pairwise distances differed very slightly (Tables S2, S3 & S4). Gaps were observed between intra- and interspecific distances in the COI, 16S rDNA and COI + 16S rDNA datasets indicating that these markers, when used individually or in combination, effectively delineated *P. canaliculata* from *P. maculata* (Fig. 5 and Table 3). The COI data indicated that *P. canaliculata* exhibited the highest intraspecific genetic divergence, which ranged from 0.0% to 4.9%. However, no variation was observed in *P. maculata* whose maximum pairwise genetic distance was 0.0%, indicating the presence of a single haplotype. The 16S rDNA marker showed little intraspecific genetic divergence in *P. canaliculata* (0.0% to 0.6%) in contrast to the COI marker. The intraspecific genetic distances observed in *P. maculata* from the 16S rDNA showed somewhat greater variation than those generated by the COI dataset; 49% of the *P. maculata* intraspecific distances showed a sequence variation of 0.2%. Concatenation of COI and 16S rDNA barcoding markers generated the highest variation (0.0% to 1.0%) among the *P. maculata* specimens where the highest intraspecific distances were attributed to the COI-16S rDNA incongruent individual (GC7). When the COI and 16S rDNA species identification were congruent, the COI + 16S rDNA dataset generated intraspecific distances in *P. canaliculata* that were intermediate of both individual marker counterparts (0.0% to 3.0%).

**Table 2:** Composition of characters in the COI, 16S rDNA and COI + 16S rDNA aligned datasets.

**Loci**	**Sequence length (bp)**	**Variable sites**	
		**Parsimony informative**	**Singleton**
COI	612	73	4
16S rDNA	472	13	0
COI + 16S rDNA	1,084	86	4

The COI, 16S rDNA and COI + 16S rDNA datasets generated significant gaps between intra- and interspecific distances. The first barcoding gap criteria (Meier et al., 2006; Meier, Zhang & Ali, 2008) generated the widest range of distance between the lowest interspecific and highest intraspecific distances and hence, the greatest barcoding gap for the COI dataset (6.06%) as compared to the 16S rDNA dataset (1.54%) and COI + 16S rDNA dataset (3.02%). Similar results were obtained when 5% of the largest intraspecific distances and 5% of the smallest interspecific distances were excluded (90% pairwise genetic distances) (Fig. 5). However, based on the second barcoding gap criteria (Hebert et al., 2004), the 16S rDNA exhibited the best performance, generating a 12-fold barcoding gap based on the ratio of the mean inter- and intraspecific distances. The COI dataset generated an approximate 9-fold barcoding gap. Similarly, the COI + 16S rDNA dataset generated a 9-fold barcoding gap. Thus, the concatenated COI + 16S rDNA dataset did not improve upon the performance of the COI and 16S rDNA markers in either criterion.

**Figure 6:**
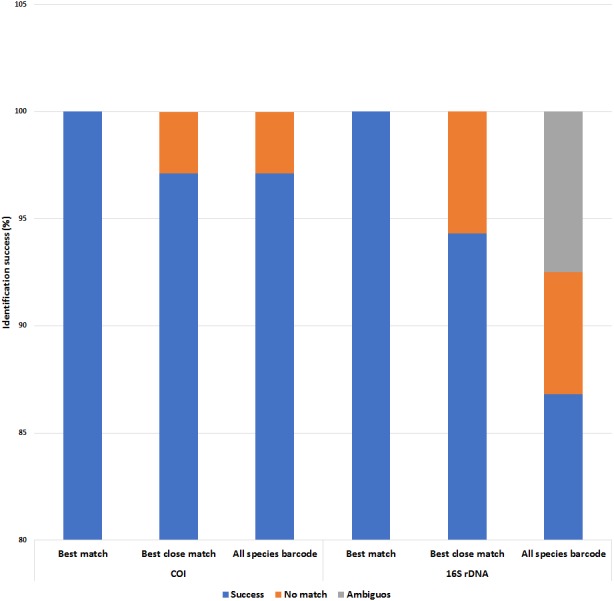
Identification success of the COI and 16S rDNA barcoding markers based on the ‘best match’, ‘best close match’ and ‘all species barcode’ approaches. A 5% intraspecific cut-off value was used for ‘best close match’ and ‘all species barcode’.

Both COI and 16S rDNA barcoding markers achieved 100% success for the ‘best match’ analyses where all species were correctly identified (Fig. 6 and Tables S5 & S6). However, when a 5% intraspecific cut-off value was implemented as a threshold value for the ‘best close match’ analysis, the success rate decreased to 97.1% and 94.3% for the COI and 16S rDNA datasets respectively. The proportion of sequences without a match for the COI dataset was due to two native *P. scalaris* (EU528506 and FJ710316) sequences having successful matches outside of the threshold (5.12%) (Table S5). As for the 16S rDNA dataset, the proportion of sequences without a successful match within the threshold (0.86%) was due to three native sequences; one *P. maculata* (FJ710228) and two *P. scalaris* (FJ710240 and FJ710241). The threshold values were also implemented for the ‘all species barcode’ analyses. The COI dataset exhibited a similar performance as in the ‘best close match’ criteria with 97.1% correct identifications. Similarly, the remaining 2.89% with no matches within the threshold comprised the two *P. scalaris* (EU528506 and FJ710316) sequences. The 16S rDNA dataset had a lower success rate at 86.8% correct identifications. The remaining proportions were represented by 7.5% ambiguous identifications (two native *P. lineata* and *P. paludosa* sequences from Table 1) and 5.7% sequences with no match within the threshold (FJ710228, FJ710240 and FJ710241).

**Table 3:** Genetic distance of *Pomacea* specimens using COI, 16S rDNA and COI + 16S rDNA aligned datasets. Pairwise intra- and interspecific genetic distances were evaluated based on the K2P evolutionary model.

**Taxa**	**Genetic Distance (%)**								
	**COI**			**16S rDNA**			**COI + 16S rDNA**		
	**Min**	**Mean**	**Max**	**Min**	**Mean**	**Max**	**Min**	**Mean**	**Max**
**Intraspecific**							
*P. canaliculata*	0.0	2.4	4.9	0.0	0.3	0.6	0.0	1.5	3.0
*P. maculata*	0.0	0.0	0.0	0.0	0.1	0.2	0.0	0.1	1.0
							
**Interspecific**	11.0	11.2	11.9	2.2	2.4	2.6	6.1	7.2	7.6

## Discussion

### Comparison of mitochondrial COI and 16S rDNA genes as barcoding markers

In Peninsular Malaysia, *P. canaliculata* and *P. maculata* have a widespread distribution but these two cryptic species are morphologically similar with limited species-specific phenotypic characteristics which complicates species identification. Mitochondrial COI has shown a distinct separation in species identification in Malaysia (Rama Rao et al., 2018) but relying on only one barcoding marker may sometimes lead to undetected misidentification (Vences et al., 2005). Mitochondrial markers have several characteristics that fulfil the DNA barcoding criteria (Arif et al., 2011; Brown, George & Allan, 1979), and so we aimed to assess the effectiveness of two mitochondrial markers, COI and 16S rDNA, as DNA barcodes for *P. canaliculata* and *P. maculata*. Our results confirmed the presence of two highly invasive apple snails, *P. canaliculata* and *P. maculata*, in Peninsular Malaysia using both COI and 16S rDNA mitochondrial barcoding markers with good barcoding results.

Portions of the COI and 16S rDNA genes used in this study are common DNA barcoding markers and have proven to be amplifiable in various species (Mc Donnell et al., 2010; Lim et al., 2012; Xia et al., 2011). The ability of the universal primers to anneal to the conserved COI and 16S rDNA barcoding regions and successfully amplify them indicate that these markers are applicable in *P. canaliculata* and *P. maculata*. The amplification success obviates the need of optimized barcoding primers as seen in other families of Caenogastropoda (Turritellidae, Planaxidae and Naticidae) where incompatible primer sites might have led to amplification failure with these universal COI primer sets (Sun et al., 2011). The bidirectional sequencing of the mitochondrial barcoding regions in all 40 specimens had little to no ambiguous bases, thus making subsequent analyses of the specimens’ sequence reads reliable.

Phylogenetic analyses of both COI and 16S rDNA barcoding markers grouped the apple snails in this study into two genetically distinct clades representing the two species of *Pomacea* found in Malaysia. The phylogeny reconstructed with both barcoding markers grouped each species in monophyly, which is a typical indication of successful barcoding (Hebert et al., 2004; Meier et al., 2006). The species discrimination provided by the barcoding markers confirmed previous morphological and molecular COI data of both *P. canaliculata* and *P. maculata* as two widespread species of non-native apple snails in Malaysia (Arfan et al., 2014; Arfan et al., 2016; Rama Rao et al., 2018). Both COI and 16S rDNA phylogenetic trees possessed similar topologies confirming clear phylogenetic signals for discriminating between these two species of apple snails (Figs. 2 & 3). Although trees were multifurcated within species, with partially resolved phylogenies as depicted by polytomies, the separation was sufficient for delineating species boundaries. Within the *P. canaliculata* clade, both markers showed similar resolving power where two divergent clades contained the specimens in this study. However, for *P. maculata*, the 16S rDNA marker resolved the specimens into two divergent clades in the ML analysis. In contrast, the COI phylogenetic data of *P. maculata* revealed a single haplotype with intraspecific distances of zero among all sampled individuals (Table 3 & Table S2). Thus, the 16S rDNA gene could potentially serve as a promising marker to study the genetic diversity of apple snails, particularly, *P. maculata*.

Inter- and intraspecific genetic variations in the COI and 16S rDNA barcoding markers was assessed to determine the extent of barcoding variation to delineate species boundaries of *P. canaliculata* and *P. maculata*. Both barcoding markers possessed relatively low intraspecific variation in both *P. canaliculata* and *P. maculata* as well as high interspecific variation with no overlap in the distribution of inter- and intraspecific genetic distances. Li, Bian & Zhao (2013) reported similar intraspecific distances for *P. canaliculata* in China and the United States generated by the 16S rDNA marker but slightly wider interspecific distance between *P. canaliculata* and *P. maculata* (2.3 to 3.1%). Based on the proposed definition of ‘barcoding gap’ by Meier et al. (2006) and Meier, Zhang & Ali (2008), obvious barcoding gaps were observed in the datasets of both COI and 16S rDNA markers, where the lowest interspecific distance was higher than the highest intraspecific distance (Table 3). However, by the ‘10-fold rule’ proposed by Hebert et al. (2004), the barcoding gap generated by the COI marker failed to produce a gap that was 10 times the intraspecific variation. In addition, a significant proportion (52.1%) of the intraspecific distances generated by the COI *P. canaliculata* sequences were greater than the 2.2% intraspecific reference threshold (Ratnasingham & Hebert, 2013), which further hampers its barcoding suitability. In terms of the second barcoding gap criterion, the 16S rDNA marker was better at delineating species boundaries for the specimens in this study.

Variable and parsimonious sites are important determinants of phylogenetic signals which in turn could help to discriminate species (López-Giráldez, Moeller & Townsend, 2013). The aligned datasets for both barcoding regions of COI and 16S rDNA showed that the COI region contained more variation (Table 2). The results in the current study differed from the study conducted by Hayes, Cowie & Thiengo (2009), where a concatenated dataset of three nuclear and two mitochondrial markers showed that the 16S rDNA marker contained highest proportion of variant and parsimony informative characters followed by the COI marker. Although percentage variation was considerably lower than that of Hayes, Cowie & Thiengo (2009), variation in the 16S rDNA region was sufficient to delineate species boundaries effectively. All specimen sequences in our study were successfully identified with no ambiguity in the ‘best match’, ‘best close match’ and ‘all species barcode’ analyses (Fig. 6). When reference sequences of conspecifics and congeners were considered, the COI barcoding marker performed better than the 16S rDNA barcoding marker. Lower success rates of the 16S rDNA marker are due to several GenBank sequences that did not meet the rigorous standards of the DNA similarity-based species delineation, possibly owing to insufficient sequences being employed for the analyses. The goal of this study was to prioritize identification of *P. canaliculata* and *P. maculata* within Peninsular Malaysia, so results for these two species can be upheld. As more query and reference16S rDNA sequences appear in Genbank, the potential for species delineation in *Pomacea* using this marker will be better established.

### Concatenated COI + 16S rDNA dataset

As outlined thus far, the COI and 16S rDNA markers possess desirable barcoding properties, but would concatenation of both markers provide a more powerful molecular tool, possessing the strengths of both COI and 16S rDNA portions, for resolving the taxonomic positions and barcoding of apple snails? In the distance-based analyses, the performance of the COI + 16S rDNA dataset was moderate and did not outperform the single marker counterparts. Thus, barcoding of apple snails with either COI or 16S rDNA may be sufficient. The phylogenetic reconstruction of the COI + 16S rDNA dataset confirmed the identification and discrimination of *Pomacea* spp. found in Peninsular Malaysia in two strongly supported clades of *P. canaliculata* and *P. maculata* (Fig. 4). The discrimination provided by the COI + 16S rDNA dataset tallies with the COI phylogeny since the COI constituted a larger portion (56.5%) of the concatenated region. One notable difference was the early divergence of an individual apple snail (GC7) from the *P. canaliculata* clade in the concatenated dataset. This difference arose from the taxonomic ambiguity in species assignment of this individual where the species identities from the COI and 16S rDNA mitochondrial phylogenies were incongruent (Figs. 2 and 3).

### COI-16S rDNA mitochondrial incongruence

The linked property of mitochondrial genes suggests that phylogenetic trees generated by both barcoding markers are expected to have similar topologies with concordant identification for all specimens. We briefly address why phylogenetic species delineation by one mitochondrial marker could be incongruent with species delineation by another marker. Mitochondrial heteroplasmy, which is characterised by the coexistence of multiple distinct mitochondrial haplotypes within a single organism as a result of somatic mutations or paternal leakage of mitochondrial DNA (Chinnery et al., 2000; White et al., 2008; Campos-Soto, Torres-Pérez & Solari, 2015) may cause phylogenetic incongruences. Paternal leakage occurs when paternal mitochondrial DNA enters the cytoplasm of the oocyte, resulting in paternal and maternal mitochondrial genes in the offspring (White et al., 2008). Paternal leakage is not confined to intraspecific relationships (Sherengul, Kondo & Matsuura, 2007; Ujvari, Dowton & Madsen, 2007) but extend to interspecific relationships where several interspecific paternal leakages derived heteroplasmy cases have been reported in great tits, cicadas, fruit flies and partridges (Dokianakis & Ladoukakis, 2014; Gandolfi et al., 2017; Ciborowski et al., 2007; Kvist et al., 2003). Interspecific paternal leakage might explain the observed incongruence because hybridization between *P. canaliculata* and *P. maculata* has been reported in Japan (Matsukura et al., 2013), and occurs in several parts in Peninsular Malaysia where the species co-occur (A Kannan et al., 2019, unpublished data). Another event in which mitochondrial incongruences occur is the integration of mitochondrial genes into the nuclear genome (Zhang & Hewitt, 1996). The nuclear copies of the inserted mitochondrial gene have slower evolution rates when compared to their mitochondrial counterparts and thus confound phylogenetic findings. However, the likelihood of this is low because the mitochondrial gene copies are most likely in abundance and could mask the detection of the nuclear copies (Scheffler, 2001).

### Efficiency of COI and 16S rDNA markers for barcoding of gastropods

Species discrimination success in our study has replicated the successful use of barcoding markers in studies involving other species of Caneogastropoda. The COI interspecific variation obtained between *P. canaliculata* and *P. maculata* for species-level discrimination was considered sufficient in our study since it is comparable to the reported average COI interspecific divergences (11.1%) within the phylum Mollusca (Hebert et al., 2003). The COI barcoding marker has provided sufficient resolution in species discrimination of several medically important freshwater snails in the family Bithyniidae where 9 out of 10 studied species were successfully identified (Kulsantiwong et al., 2013). Similarly, a 10-fold barcoding gap was not obtained with high intraspecific distances reported in *Wattebedia crosseana* (4.9%), which explained the formation of two phylogenetic sequence clusters as in *P. canaliculata* in our study. Despite having several flaws, the performance of the COI marker in our study was better since significant barcoding gaps were obtained between the intra- and interspecific distances. However, our study mainly focused on *P. canaliculata* and *P. maculata* and an accurate comparison will be obtained with increased taxon sampling of congeneric specimens of *Pomacea*. The utility of the COI barcoding marker also extends to marine species of Caneogastropoda, where the COI barcoding region successfully characterized 13 families (Sun et al., 2011).

Although the 16S rDNA marker is becoming increasingly common in establishing phylogenetic relationships among gastropods owing to its highly variable nature (Thollesson, 1999; Aguilera-Muñoz, Lafarga-Cruz & Gallardo-Escárate, 2019), limited studies have focused on its barcoding efficiency. The results in our study reported the promising use of the 16S rDNA marker as a barcode for ampullariids studied which corresponds with the monophylies recovered by Jørgensen, Kristensen & Madsen (2008) and Hayes, Cowie & Thiengo (2009). However, the utility of the 16S rDNA as a barcoding marker may sometimes be hindered when utilized in a wider context; for instance, in a study involving 40 species of caenogastropods, the 16S marker exhibited poor distance and tree-based barcoding performances and could only be delineated via a character-based barcoding approach (Zou et al., 2011). Other 16S rDNA barcoding studies have instead incorporated additional genes to improve barcoding efficiency; (i) mitochondrial 12S rDNA marker and the standard animal COI barcode in barcoding the egg capsules of several marine caenogastropods (Puillandre et al., 2009) and (ii) the inclusion of nuclear loci to complement the mitochondrial locus-based barcoding approach (Zou, Li & Kong, 2011; Zielske & Haase, 2015). Zielske & Haase (2015) reported inconsistencies in the mitochondrial (COI + 16S rDNA) and the nuclear internal transcribed spacer-2 (ITS-2) phylogenies for two well defined species of freshwater gastropods, *Hemistomia cockerelli* and *H. fabrorum* which may reflect the evolutionary consequence of hybridization.

The multi-loci barcoding approach is gaining significant attention in population genetics because multiple loci from the nuclear genome with different evolutionary rates provide a more comprehensive approach that enable comparison of several inferred genealogies to investigate the evolutionary history of diverging populations. This is an important notion in elucidating evolutionary hypotheses such as introgressive hybridization between closely related species (Good et al., 2008). Our study focused on a single linked locus approach, thus, is restricted to a single genealogy out of several evolutionary possible genealogies and may not necessarily reflect the actual population history. The lack of nuclear markers in our study prevents us from further addressing the COI-16S rDNA incongruence as a consequence of possible introgressive hybridization driven heteroplasmy from an empirical perspective. Several studies have utilized nuclear markers such as mobile elements and housekeeping genes to infer phylogenies for molecular evidences of hybridization (Sequeira et al., 2011; Roos et al., 2011; Matsukura et al., 2013; Hirano et al., 2019) and such approaches could be employed in the future to investigate if hybridization has occurred between *P. canaliculata* and *P. maculata* in the peninsula.

## Conclusions

Based on the findings of this study, both mitochondrial COI and 16S rDNA barcoding markers were able to successfully barcode the specimens in our study and hence, delineate species boundaries of *P. canaliculata* and *P. maculata*. Although both markers are suitable and reliable markers for *Pomacea* species identification, the application of these markers may vary in performance, for instance, the 16S rDNA marker showed better phylogenetic resolution and ‘barcoding gap’ folds whereas the highly variant COI barcoding region with a plethora of sequences in the NCBI database was the better marker in similarity-based identifications. Considering all the analyses, the COI marker is an ideal DNA barcode for species discrimination of *Pomacea*. Additionally, the one incongruence reported in our study supports previous morphological evidence of interspecific hybridization in Peninsular Malaysia. The use of either mitochondrial barcoding marker in this study will complement nuclear markers to assess hybridization studies in Peninsular Malaysia which has important ecological consequences.

##  Supplemental Information

10.7717/peerj.8755/supp-1 Table S1 List of *Pomacea* spp. COI reference sequences used in this study with their corresponding GenBank and BOLD accession numbers. A total of 29 COI sequences were retrieved from 13 countries A total of 29 COI sequences were retrieved from 13 countries.

10.7717/peerj.8755/supp-2 Table S2 Genetic divergence analysis of the COI marker based on pairwise alignment of all 40 Pomacea specimens. The genetic distances were computed using the K2P (below diagonal) and uncorrected p-distance (above diagonal) The genetic distances were computed using the K2P (below diagonal) and uncorrected p-distance (above diagonal).

10.7717/peerj.8755/supp-3 Table S3 Genetic divergence analysis of the 16S rDNA marker based on pairwise alignment of all 40 Pomacea specimens The genetic distances were computed using the K2P (below diagonal) and uncorrected p-distance (above diagonal).

10.7717/peerj.8755/supp-4 Table S4 Genetic divergence analysis of the COI + 16S rDNA dataset based on pairwise alignment of all 40 Pomacea specimens The genetic distances were computed using the K2P (below diagonal) and uncorrected p-distance (above diagonal).

10.7717/peerj.8755/supp-5 Table S5 Summary of COI sequence identification via similarity DNA-based distance approaches ‘Best match’, ‘best close match’ and ‘all species barcode’ were computed using the K2P model with a minimum of 300 bp overlap in TaxonDNA Species Identifier v1.7.7. Threshold values was set where 95% of all intraspecific distances were found (Threshold value: 5.12%).

10.7717/peerj.8755/supp-6 Table S6 Summary of 16S rDNA sequence identification via similarity DNA-based distance approaches ‘Best match’, ‘best close match’ and ‘all species barcode’ were computed using the K2P model with a minimum of 300 bp overlap in TaxonDNA Species Identifier v1.7.7. Threshold values was set where 95% of all intraspecific distances were found (Threshold: 0.86 %).

10.7717/peerj.8755/supp-7 Supplemental Information 7 GenBank COI sequences: MN623417–MN623440

10.7717/peerj.8755/supp-8 Supplemental Information 8 GenBank 16SrDNA sequences: MN623441–MN623480

## Additional Information and Declarations

### Field Study Permissions

The following information was supplied relating to field study approvals (i.e., approving body and any reference numbers): Sample collection in Taman Wetland Putrajaya with permission from Environmental, Lake and Wetland Division [Reference number: PPj/R/A/TWH/69(14)]. No specific permission was required for other locations as it did not involve any endangered or protected species.

### DNA Deposition

The following information was supplied regarding the deposition of DNA sequences: The 64 new sequences are available at GenBank: MN623417–MN623480.

### Data Availability

The following information was supplied regarding data availability: The raw data are available in the Supplemental Files.
