# Supplementary material for: The efficiency of universal mitochondrial DNA barcodes for species discrimination of Pomacea canaliculata and Pomacea maculata
Source: PeerJ. 2020 Apr 1;8:e8755. doi: 10.7717/peerj.8755 (PMC7127494; doi:10.7717/peerj.8755)
Supplement: Table S1 — A total of 29 COI sequences were retrieved from 13 countries. [file peerj-08-8755-s001.docx]

**Table S1: List of *Pomacea* spp. COI reference sequences used in this study with their corresponding GenBank and BOLD accession numbers.** A total of 29 COI sequences were retrieved from 13 countries.

| **Sequence ID/ Species** | **Location** | **GenBank Accession Number** | | **BOLD Accession Number** |
| --- | --- | --- | --- | --- |
| *P. canaliculata* | Malaysia | MG230755 | GBML12884-19 | |
|  | Singapore | KY081757 | - | |
|  | Indonesia | KY574007 | GBMIN109442-17 | |
|  | China | FJ946820 | GBMLG8773-09 | |
|  |  | FJ946823 | GBMLG8770-09 | |
|  | Hong Kong | KT313034 | GBMIN109430-17 | |
|  | Japan | AB433773 | GBMLG8300-09 | |
|  | Philippines | EU528483 | GBMLG8612-09 | |
|  | Chile | KX965671 | GBMIN109443-17 | |
|  | USA | EF514982 | GBMLG3515-07 | |
|  | Argentina | AB728574 | GBMLG13355-13 | |
|  |  | FJ710314 | GBMLG9050-10 | |
|  |  | FJ710315 | GBMLG9049-10 | |
|  |  | EU528529 | GBMLG8566-09 | |
|  | Uruguay | FJ710313 | GBMLG9051-10 | |
| *P. maculata* | Malaysia | MG230787 | GBML12982-19 | |
|  | China | FJ946828 | GBMLG8765-09 | |
|  | Japan | AB433781 | GBMLG8292-09 | |
|  | Spain | GU236491 | GBMIN3119-12 | |
|  | USA | JX845573 | GBMIN3119-12 | |
|  | Brazil | EU528559 | GBMLG8536-09 | |
| *P. lineata* | Brazil | FJ710309 | GBMLG9055-10 | |
|  |  | FJ710310 | GBMLG9054-10 | |
|  |  | FJ710311 | GBMLG9053-10 | |
| *P. paludosa* | USA | EF514960 | GBMLG7607-09 | |
|  |  | EU528590 | GBMLG8505-09 | |
|  |  | EU528591 | GBMLG8504-09 | |
| *P. scalaris* | Argentina | EU528506 | GBMLG8589-09 | |
|  | Brazil | FJ710316 | GBMLG9048-10 | |

‘-‘ indicate sequence that is not unavailable in the BOLD public repository database
