# Supplementary material for: The efficiency of universal mitochondrial DNA barcodes for species discrimination of Pomacea canaliculata and Pomacea maculata
Source: PeerJ. 2020 Apr 1;8:e8755. doi: 10.7717/peerj.8755 (PMC7127494; doi:10.7717/peerj.8755)
Supplement: Table S5 — ‘Best match’, ‘best close match’ and ‘all species barcode’ were computed using the K2P model with a minimum of 300 bp overlap in TaxonDNA Species Identifier v1.7.7. Threshold values was set where 95% of all intraspecific distances were found (Threshold value: 5.12%). [file peerj-08-8755-s005.docx]

**Table S5: Summary of COI sequence identification via similarity DNA-based distance approaches**. ‘Best match’, ‘best close match’ and ‘all species barcode’ were computed using the K2P model with a minimum of 300 bp overlap in TaxonDNA Species Identifier v1.7.7. Threshold values was set where 95% of all intraspecific distances were found (Threshold value: 5.12%).

| **Query** | **First Conspecific match** | **Distance** | **Overlap** | **Closest Allospecific match** | **Distance** | **Overlap** | **Other matches** | **Remarks** | **Best Match** | **Best Close Match** | **All Species Barcode** |
| --- | --- | --- | --- | --- | --- | --- | --- | --- | --- | --- | --- |
| *P. canaliculata* (SJ2) | *P. canaliculata* (SJ5) | 0.0 | 611 | *P. maculata* (gi:414420913) | 10.0 | 611 | *P. canaliculata* (SJ5) and 7 others | Successful match at 0.0% (within threshold) | Correct | Correct | Correct |
| *P. canaliculata* (gi:1124149291) | *P. canaliculata* (gi:1389533307) | 0.16 | 611 | *P. maculata* (gi:190693817) | 10.4 | 611 | *P. canaliculata* (gi:1389533307) and 13 others | Successful match at 0.16% (within threshold) | Correct | Correct | Correct |
| *P. canaliculata* (gi:1168026905) | *P. canaliculata* (gi:257783391) | 1.15 | 611 | *P. maculata* (gi:414420913) | 9.79 | 611 | *P. canaliculata* (gi:257783391) | Successful match at 1.15% (within threshold) | Correct | Correct | Correct |
| *P. canaliculata* (SJ5) | *P. canaliculata* (SJ2) | 0.0 | 611 | *P. maculata* (gi:414420913) | 10.0 | 611 | *P. canaliculata* (SJ2) and 7 others | Successful match at 0.0% (within threshold) | Correct | Correct | Correct |
| *P. canaliculata* (gi:1227151841) | *P. canaliculata* (gi:1389533307) | 0.16 | 611 | *P. maculata* (gi:190693817) | 10.0 | 611 | *P. canaliculata* (gi:1389533307) and 15 others | Successful match at 0.16% (within threshold) | Correct | Correct | Correct |
| *P. canaliculata* (SJ7) | *P. canaliculata* (SJ2) | 0.0 | 611 | *P. maculata* (gi:414420913) | 10.0 | 611 | *P. canaliculata* (SJ2) and 7 others | Successful match at 0.0% (within threshold) | Correct | Correct | Correct |
| *P. canaliculata* (gi:1389533307) | *P. canaliculata* (gi:151548795) | 0.0 | 611 | *P. maculata* (gi:190693817) | 10.2 | 611 | *P. canaliculata* (gi:151548795) and 14 others | Successful match at 0.0% (within threshold) | Correct | Correct | Correct |
| *P. canaliculata* (SJ13) | *P. canaliculata* (SJ2) | 0.0 | 611 | *P. maculata* (gi:414420913) | 10.0 | 611 | *P. canaliculata* (SJ2) and 7 others | Successful match at 0.0% (within threshold) | Correct | Correct | Correct |
| *P. canaliculata* (SJ14) | *P. canaliculata* (SJ2) | 0.0 | 611 | *P. maculata* (gi:414420913) | 10.0 | 611 | *P. canaliculata* (SJ2) and 7 others | Successful match at 0.0% (within threshold) | Correct | Correct | Correct |
| *P. canaliculata* (gi:151548795) | *P. canaliculata* (gi:1389533307) | 0.0 | 611 | *P. maculata* (gi:190693817) | 10.2 | 611 | *P. canaliculata* (gi:1389533307) and 14 others | Successful match at 0.0% (within threshold) | Correct | Correct | Correct |
| *P. canaliculata* (LL2) | *P. canaliculata* (gi:1389533307) | 0.0 | 611 | *P. maculata* (gi:190693817) | 10.2 | 611 | *P. canaliculata* (gi:1389533307) and 14 others | Successful match at 0.0% (within threshold) | Correct | Correct | Correct |
| *P. canaliculata* (gi:190693665) | *P. canaliculata* (gi:238683561) | 0.0 | 611 | *P. maculata* (gi:190693817) | 10.78 | 611 | *P. canaliculata* (gi:238683561) | Successful match at 0.0% (within threshold) | Correct | Correct | Correct |
| *P. canaliculata* (gi:190693757) | *P. canaliculata* (gi:1389533307) | 0.16 | 611 | *P. maculata* (gi:190693817) | 10.2 | 611 | *P. canaliculata* (gi:1389533307) and 13 others | Successful match at 0.16% (within threshold) | Correct | Correct | Correct |
| *P. canaliculata* (LL5) | *P. canaliculata* (gi:1227151841) | 0.66 | 611 | *P. maculata* (gi:190693817) | 10.92 | 611 | *P. canaliculata* (gi:923144654) | Successful match at 0.66% (within threshold) | Correct | Correct | Correct |
| *P. canaliculata* (LL5) | *P. canaliculata* (gi:1389533307) | 0.0 | 611 | *P. maculata* (gi:190693817) | 10.2 | 611 | *P. canaliculata* (gi:1389533307) and 14 others | Successful match at 0.0% (within threshold) | Correct | Correct | Correct |
| *P. canaliculata* (PJ1) | *P. canaliculata* (SJ2) | 0.0 | 611 | *P. maculata* (gi:414420913) | 10.0 | 611 | *P. canaliculata* (SJ2) and 7 others | Successful match at 0.0% (within threshold) | Correct | Correct | Correct |
| *P. canaliculata* (PJ5) | *P. canaliculata* (SJ2) | 0.0 | 611 | *P. maculata* (gi:414420913) | 10.0 | 611 | *P. canaliculata* (SJ2) and 7 others | Successful match at 0.0% (within threshold) | Correct | Correct | Correct |
| *P. canaliculata* (gi:226442464) | *P. canaliculata* (gi:1389533307) | 0.0 | 611 | *P. maculata* (gi:190693817) | 10.2 | 611 | *P. canaliculata* (gi:1389533307) and 14 others | Successful match at 0.0% (within threshold) | Correct | Correct | Correct |
| *P. canaliculata* (PJ13) | *P. canaliculata* (SJ2) | 0.0 | 611 | *P. maculata* (gi:414420913) | 10.0 | 611 | *P. canaliculata* (SJ2) and 7 others | Successful match at 0.0% (within threshold) | Correct | Correct | Correct |
| *P. canaliculata* (gi:238683555) | *P. canaliculata* (gi:1389533307) | 0.0 | 611 | *P. maculata* (gi:190693817) | 10.09 | 611 | *P. canaliculata* (gi:1389533307) and 14 others | Successful match at 0.0% (within threshold) | Correct | Correct | Correct |
| *P. canaliculata* (gi:238683561) | *P. canaliculata* (gi:190693665) | 0.0 | 611 | *P. maculata* (gi:190693817) | 10.68 | 611 | *P. canaliculata* (gi:190693665) | Successful match at 0.0% (within threshold) | Correct | Correct | Correct |
| *P. canaliculata* (PJ14) | *P. canaliculata* (gi:1389533307) | 0.0 | 611 | *P. maculata* (gi:190693817) | 10.2 | 611 | *P. canaliculata* (gi:1389533307) and 14 others | Successful match at 0.0% (within threshold) | Correct | Correct | Correct |
| *P. canaliculata* (PJ16) | *P. canaliculata* (gi:1389533307) | 0.0 | 611 | *P. maculata* (gi:190693817) | 10.2 | 611 | *P. canaliculata* (gi:1389533307) and 14 others | Successful match at 0.0% (within threshold) | Correct | Correct | Correct |
| *P. canaliculata* (gi:257783391) | *P. canaliculata* (gi:1168026905) | 1.15 | 611 | *P. maculata* (gi:190693817) | 10.58 | 611 | *P. canaliculata* (gi:1168026905) | Successful match at 1.15% (within threshold) | Correct | Correct | Correct |
| *P. canaliculata* (gi:257783393) | *P. canaliculata* (gi:1168026905) | 1.83 | 611 | *P. maculata* (gi:190693817) | 10.19 | 611 | *P. canaliculata* (gi:1168026905) | Successful match at 1.83% (within threshold) | Correct | Correct | Correct |
| *P. canaliculata* (gi:257783395) | *P. canaliculata* (gi:1389533307) | 0.0 | 611 | *P. maculata* (gi:190693817) | 10.2 | 611 | *P. canaliculata* (gi:1389533307) and 14 others | Successful match at 0.0% (within threshold) | Correct | Correct | Correct |
| *P. canaliculata* (PJ45) | *P. canaliculata* (SJ2) | 0.0 | 611 | *P. maculata* (gi:414420913) | 10.0 | 611 | *P. canaliculata* (SJ2) and 7 others | Successful match at 0.0% (within threshold) | Correct | Correct | Correct |
| *P. canaliculata* (PJ2) | *P. canaliculata* (gi:1389533307) | 0.0 | 611 | *P. maculata* (gi:190693817) | 10.2 | 611 | *P. canaliculata* (gi:1389533307) and 14 others | Successful match at 0.0% (within threshold) | Correct | Correct | Correct |
| *P. canaliculata* (GC6) | *P. canaliculata* (gi:1389533307) | 0.0 | 611 | *P. maculata* (gi:190693817) | 10.2 | 611 | *P. canaliculata* (gi:1389533307) and 14 others | Successful match at 0.0% (within threshold) | Correct | Correct | Correct |
| *P. canaliculata* (GC8) | *P. canaliculata* (gi:1389533307) | 0.0 | 611 | *P. maculata* (gi:190693817) | 10.2 | 611 | *P. canaliculata* (gi:1389533307) and 14 others | Successful match at 0.0% (within threshold) | Correct | Correct | Correct |
| *P. canaliculata* (GC18) | *P. canaliculata* (gi:1389533307) | 0.0 | 611 | *P. maculata* (gi:190693817) | 10.2 | 611 | *P. canaliculata* (gi:1389533307) and 14 others | Successful match at 0.0% (within threshold) | Correct | Correct | Correct |
| *P. canaliculata* (GC20) | *P. canaliculata* (gi:1389533307) | 0.0 | 611 | *P. maculata* (gi:190693817) | 10.2 | 611 | *P. canaliculata* (gi:1389533307) and 14 others | Successful match at 0.0% (within threshold) | Correct | Correct | Correct |
| *P. canaliculata* (gi:429535651) | *P. canaliculata* (gi:1168026905) | 2.16 | 611 | *P. maculata* (gi:414420913) | 10.54 | 611 | *P. canaliculata* (gi:1168026905) | Successful match at 2.16% (within threshold) | Correct | Correct | Correct |
| *P. canaliculata* (LL6) | *P. canaliculata* (gi:1389533307) | 0.0 | 611 | *P. maculata* (gi:190693817) | 10.2 | 611 | *P. canaliculata* (gi:1389533307) and 14 others | Successful match at 0.0% (within threshold) | Correct | Correct | Correct |
| *P. canaliculata* (gi:923144654) | *P. canaliculata* (gi:1389533307) | 0.0 | 611 | *P. maculata* (gi:190693817) | 10.24 | 611 | *P. canaliculata* (gi:1389533307) and 14 others | Successful match at 0.0% (within threshold) | Correct | Correct | Correct |
| *P. lineata* (gi:257783383) | *P. lineata* (gi:257783385) | 1.66 | 611 | *P. maculata* (gi:190693817) | 8.23 | 611 | *P. lineata* (gi:257783385) | Successful match at 1.66% (within threshold) | Correct | Correct | Correct |
| *P. lineata* (gi:257783385) | *P. lineata* (gi:257783387) | 0.0 | 611 | *P. maculata* (gi:190693817) | 8.61 | 611 | *P. lineata* (gi:257783387) | Successful match at 0.0% (within threshold) | Correct | Correct | Correct |
| *P. lineata* (gi:257783387) | *P. lineata* (gi:257783385) | 0.0 | 611 | *P. maculata* (gi:190693817) | 8.63 | 611 | *P. lineata* (gi:257783385) | Successful match at 0.0% (within threshold) | Correct | Correct | Correct |
| *P. maculata* (AN1) | *P. maculata* (CC5) | 0.0 | 611 | *P. lineata* (gi:257783383) | 9.19 | 611 | *P. maculata* (CC5) and 22 others | Successful match at 0.0% (within threshold) | Correct | Correct | Correct |
| *P. maculata* (CC5) | *P. maculata* (AN1) | 0.0 | 611 | *P. lineata* (gi:257783383) | 9.19 | 611 | *P. maculata* (AN1) and 22 others | Successful match at 0.0% (within threshold) | Correct | Correct | Correct |
| *P. maculata* (gi:1389533367) | *P. maculata* (AN1) | 0.0 | 611 | *P. lineata* (gi:257783383) | 9.19 | 611 | *P. maculata* (AN1) and 22 others | Successful match at 0.0% (within threshold) | Correct | Correct | Correct |
| *P. maculata* (gi:190693817) | *P. maculata* (gi:414420913) | 2.68 | 611 | *P. lineata* (gi:257783383) | 8.23 | 611 | *P. maculata* (gi:414420913) | Successful match at 2.68% (within threshold) | Correct | Correct | Correct |
| *P. maculata* (AN2) | *P. maculata* (AN1) | 0.0 | 611 | *P. lineata* (gi:257783383) | 9.19 | 611 | *P. maculata* (AN1) and 22 others | Successful match at 0.0% (within threshold) | Correct | Correct | Correct |
| *P. maculata* (gi:226442480) | *P. maculata* (AN1) | 0.0 | 611 | *P. lineata* (gi:257783383) | 9.19 | 611 | *P. maculata* (AN1) and 22 others | Successful match at 0.0% (within threshold) | Correct | Correct | Correct |
| *P. maculata* (gi:238683571) | *P. maculata* (AN1) | 0.0 | 611 | *P. lineata* (gi:257783383) | 9.27 | 611 | *P. maculata* (AN1) and 22 others | Successful match at 0.0% (within threshold) | Correct | Correct | Correct |
| *P. maculata* (PJ2) | *P. maculata* (AN1) | 0.0 | 611 | *P. lineata* (gi:257783383) | 9.19 | 611 | *P. maculata* (AN1) and 22 others | Successful match at 0.0% (within threshold) | Correct | Correct | Correct |
| *P. maculata* (gi:226442480) | *P. maculata* (AN1) | 0.0 | 611 | *P. lineata* (gi:257783383) | 9.19 | 611 | *P. maculata* (AN1) and 22 others | Successful match at 0.0% (within threshold) | Correct | Correct | Correct |
| *P. maculata* (AN3) | *P. maculata* (AN1) | 0.0 | 611 | *P. lineata* (gi:257783383) | 9.19 | 611 | *P. maculata* (AN1) and 22 others | Successful match at 0.0% (within threshold) | Correct | Correct | Correct |
| *P. maculata* (PJ3) | *P. maculata* (AN1) | 0.0 | 611 | *P. lineata* (gi:257783383) | 9.19 | 611 | *P. maculata* (AN1) and 22 others | Successful match at 0.0% (within threshold) | Correct | Correct | Correct |
| *P. maculata* (PJ4) | *P. maculata* (AN1) | 0.0 | 611 | *P. lineata* (gi:257783383) | 9.19 | 611 | *P. maculata* (AN1) and 22 others | Successful match at 0.0% (within threshold) | Correct | Correct | Correct |
| *P. maculata* (PJ11) | *P. maculata* (AN1) | 0.0 | 611 | *P. lineata* (gi:257783383) | 9.19 | 611 | *P. maculata* (AN1) and 22 others | Successful match at 0.0% (within threshold) | Correct | Correct | Correct |
| *P. maculata* (PJ15) | *P. maculata* (AN1) | 0.0 | 611 | *P. lineata* (gi:257783383) | 9.19 | 611 | *P. maculata* (AN1) and 22 others | Successful match at 0.0% (within threshold) | Correct | Correct | Correct |
| *P. maculata* (AN4) | *P. maculata* (AN1) | 0.0 | 611 | *P. lineata* (gi:257783383) | 9.19 | 611 | *P. maculata* (AN1) and 22 others | Successful match at 0.0% (within threshold) | Correct | Correct | Correct |
| *P. maculata* (GC1) | *P. maculata* (AN1) | 0.0 | 611 | *P. lineata* (gi:257783383) | 9.19 | 611 | *P. maculata* (AN1) and 22 others | Successful match at 0.0% (within threshold) | Correct | Correct | Correct |
| *P. maculata* (GC2) | *P. maculata* (AN1) | 0.0 | 611 | *P. lineata* (gi:257783383) | 9.19 | 611 | *P. maculata* (AN1) and 22 others | Successful match at 0.0% (within threshold) | Correct | Correct | Correct |
| *P. maculata* (gi:414420913) | *P. maculata* (AN1) | 1.15 | 611 | *P. lineata* (gi:257783383) | 9.0 | 611 | *P. maculata* (AN1) and 22 others | Successful match at 1.15% (within threshold) | Correct | Correct | Correct |
| *P. maculata* (GC5) | *P. maculata* (AN1) | 0.0 | 611 | *P. lineata* (gi:257783383) | 9.19 | 611 | *P. maculata* (AN1) and 22 others | Successful match at 0.0% (within threshold) | Correct | Correct | Correct |
| *P. maculata* (GC7) | *P. maculata* (AN1) | 0.0 | 611 | *P. lineata* (gi:257783383) | 9.19 | 611 | *P. maculata* (AN1) and 22 others | Successful match at 0.0% (within threshold) | Correct | Correct | Correct |
| *P. maculata* (GC11) | *P. maculata* (AN1) | 0.0 | 611 | *P. lineata* (gi:257783383) | 9.19 | 611 | *P. maculata* (AN1) and 22 others | Successful match at 0.0% (within threshold) | Correct | Correct | Correct |
| *P. maculata* (AN5) | *P. maculata* (AN1) | 0.0 | 611 | *P. lineata* (gi:257783383) | 9.19 | 611 | *P. maculata* (AN1) and 22 others | Successful match at 0.0% (within threshold) | Correct | Correct | Correct |
| *P. maculata* (CC1) | *P. maculata* (AN1) | 0.0 | 611 | *P. lineata* (gi:257783383) | 9.19 | 611 | *P. maculata* (AN1) and 22 others | Successful match at 0.0% (within threshold) | Correct | Correct | Correct |
| *P. maculata* (CC2) | *P. maculata* (AN1) | 0.0 | 611 | *P. lineata* (gi:257783383) | 9.19 | 611 | *P. maculata* (AN1) and 22 others | Successful match at 0.0% (within threshold) | Correct | Correct | Correct |
| *P. maculata* (CC3) | *P. maculata* (AN1) | 0.0 | 611 | *P. lineata* (gi:257783383) | 9.19 | 611 | *P. maculata* (AN1) and 22 others | Successful match at 0.0% (within threshold) | Correct | Correct | Correct |
| *P. maculata* (CC4) | *P. maculata* (AN1) | 0.0 | 611 | *P. lineata* (gi:257783383) | 9.19 | 611 | *P. maculata* (AN1) and 22 others | Successful match at 0.0% (within threshold) | Correct | Correct | Correct |
| *P. paludosa* (gi:151548751) | *P. paludosa* (gi:190693881) | 0.66 | 611 | *P. lineata* (gi:257783383) | 13.68 | 611 | *P. paludosa* (gi:190693881) | Successful match at 0.66% (within threshold) | Correct | Correct | Correct |
| *P. paludosa* (gi:190693879) | *P. paludosa* (gi:190693881) | 1.33 | 611 | *P. canaliculata* (gi:1168026905) | 13.45 | 611 | *P. paludosa* (gi:190693881) | Successful match at 1.33% (within threshold) | Correct | Correct | Correct |
| *P. paludosa* (gi:190693881) | *P. paludosa* (gi:151548751) | 0.66 | 611 | *P. canaliculata* (gi:1168026905) | 13.58 | 611 | *P. paludosa* (gi:151548751) | Successful match at 0.66% (within threshold) | Correct | Correct | Correct |
| *P. scalaris* (gi:190693711) | *P. scalaris* (gi:257783397) | 5.55 | 610 | *P. canaliculata* (gi:238683555) | 15.93 | 610 | *P. scalaris* (gi:257783397) | Successful match at 5.55% (outside threshold) | Correct | No match | No match |
| *P. scalaris* (gi:257783397) | *P. scalaris* (gi:190693711) | 5.55 | 610 | *P. canaliculata* (gi:238683555) | 15.75 | 610 | *P. scalaris* (gi:190693711) | Successful match at 5.55% (outside threshold) | Correct | No match | No match |
