# Supplementary material for: The efficiency of universal mitochondrial DNA barcodes for species discrimination of Pomacea canaliculata and Pomacea maculata
Source: PeerJ. 2020 Apr 1;8:e8755. doi: 10.7717/peerj.8755 (PMC7127494; doi:10.7717/peerj.8755)
Supplement: Table S6 — ‘Best match’, ‘best close match’ and ‘all species barcode’ were computed using the K2P model with a minimum of 300 bp overlap in TaxonDNA Species Identifier v1.7.7. Threshold values was set where 95% of all intraspecific distances were found (Threshold: 0.86 %). [file peerj-08-8755-s006.docx]

**Table S6: Summary of 16S rDNA sequence identification via similarity DNA-based distance approaches**. ‘Best match’, ‘best close match’ and ‘all species barcode’ were computed using the K2P model with a minimum of 300 bp overlap in TaxonDNA Species Identifier v1.7.7. Threshold values was set where 95% of all intraspecific distances were found (Threshold: 0.86 %).

| **Query** | **First Conspecific match** | **Distance** | **Overlap** | **Closest Allospecific match** | **Distance** | **Overlap** | **Other matches** | **Remarks** | **Best Match** | **Best Close Match** | **All Species Barcode** |
| --- | --- | --- | --- | --- | --- | --- | --- | --- | --- | --- | --- |
| *P. canaliculata* (SJ2) | *P. canaliculata* (SJ5) | 0.0 | 470 | *P. lineata* (gi:257783477) | 1.73 | 467 | *P. canaliculata* (SJ5) and 7 others | Successful match at 0.0% (within threshold) | Correct | Correct | Correct |
| *P. canaliculata* (SJ5) | *P. canaliculata* (SJ2) | 0.0 | 470 | *P. lineata* (gi:257783477) | 1.73 | 467 | *P. canaliculata* (SJ2) and 7 others | Successful match at 0.0% (within threshold) | Correct | Correct | Correct |
| *P. canaliculata* (SJ7) | *P. canaliculata* (SJ2) | 0.0 | 470 | *P. lineata* (gi:257783477) | 1.73 | 467 | *P. canaliculata* (SJ2) and 7 others | Successful match at 0.0% (within threshold) | Correct | Correct | Correct |
| *P. canaliculata* (SJ13) | *P. canaliculata* (SJ2) | 0.0 | 470 | *P. lineata* (gi:257783477) | 1.73 | 467 | *P. canaliculata* (SJ2) and 7 others | Successful match at 0.0% (within threshold) | Correct | Correct | Correct |
| *P. canaliculata* (SJ14) | *P. canaliculata* (SJ2) | 0.0 | 470 | *P. lineata* (gi:257783477) | 1.73 | 467 | *P. canaliculata* (SJ2) and 7 others | Successful match at 0.0% (within threshold) | Correct | Correct | Correct |
| *P. canaliculata* (gi:167614052) | *P. canaliculata* (LL2) | 0.0 | 467 | *P. lineata* (gi:257783477) | 1.52 | 467 | *P. canaliculata* (LL2) and 14 others | Successful match at 0.0% (within threshold) | Correct | Correct | Correct |
| *P. canaliculata* (LL2) | *P. canaliculata* (gi:167614052) | 0.0 | 467 | *P. lineata* (gi:257783477) | 1.52 | 467 | *P. canaliculata* (gi:167614052) and 14 others | Successful match at 0.0% (within threshold) | Correct | Correct | Correct |
| *P. canaliculata* (LL5) | *P. canaliculata* (gi:167614052) | 0.0 | 467 | *P. lineata* (gi:257783477) | 1.52 | 467 | *P. canaliculata* (gi:167614052) and 14 others | Successful match at 0.0% (within threshold) | Correct | Correct | Correct |
| *P. canaliculata* (PJ1) | *P. canaliculata* (SJ2) | 0.0 | 470 | *P. lineata* (gi:257783477) | 1.73 | 467 | *P. canaliculata* (SJ2) and 7 others | Successful match at 0.0% (within threshold) | Correct | Correct | Correct |
| *P. canaliculata* (PJ5) | *P. canaliculata* (SJ2) | 0.0 | 470 | *P. lineata* (gi:257783477) | 1.73 | 467 | *P. canaliculata* (SJ2) and 7 others | Successful match at 0.0% (within threshold) | Correct | Correct | Correct |
| *P. canaliculata* (PJ13) | *P. canaliculata* (SJ2) | 0.0 | 470 | *P. lineata* (gi:257783477) | 1.73 | 467 | *P. canaliculata* (SJ2) and 7 others | Successful match at 0.0% (within threshold) | Correct | Correct | Correct |
| *P. canaliculata* (PJ14) | *P. canaliculata* (gi:167614052) | 0.0 | 467 | *P. lineata* (gi:257783477) | 1.52 | 467 | *P. canaliculata* (gi:167614052) and 14 others | Successful match at 0.0% (within threshold) | Correct | Correct | Correct |
| *P. canaliculata* (PJ16) | *P. canaliculata* (gi:167614052) | 0.0 | 467 | *P. lineata* (gi:257783477) | 1.52 | 467 | *P. canaliculata* (gi:167614052) and 14 others | Successful match at 0.0% (within threshold) | Correct | Correct | Correct |
| *P. canaliculata* (gi:257783481) | *P. canaliculata* (gi:167614052) | 0.0 | 467 | *P. lineata* (gi:257783477) | 1.52 | 467 | *P. canaliculata* (gi:167614052) and 14 others | Successful match at 0.0% (within threshold) | Correct | Correct | Correct |
| *P. canaliculata* (PJ20) | *P. canaliculata* (gi:167614052) | 0.0 | 467 | *P. lineata* (gi:257783477) | 1.52 | 467 | *P. canaliculata* (gi:167614052) and 14 others | Successful match at 0.0% (within threshold) | Correct | Correct | Correct |
| *P. canaliculata* (PJ29) | *P. canaliculata* (gi:167614052) | 0.0 | 467 | *P. lineata* (gi:257783477) | 1.52 | 467 | *P. canaliculata* (gi:167614052) and 14 others | Successful match at 0.0% (within threshold) | Correct | Correct | Correct |
| *P. canaliculata* (PJ45) | *P. canaliculata* (SJ2) | 0.0 | 470 | *P. lineata* (gi:257783477) | 1.73 | 467 | *P. canaliculata* (SJ2) and 7 others | Successful match at 0.0% (within threshold) | Correct | Correct | Correct |
| *P. canaliculata* (GC6) | *P. canaliculata* (gi:167614052) | 0.0 | 467 | *P. lineata* (gi:257783477) | 1.52 | 467 | *P. canaliculata* (gi:167614052) and 14 others | Successful match at 0.0% (within threshold) | Correct | Correct | Correct |
| *P. canaliculata* (GC8) | *P. canaliculata* (gi:167614052) | 0.0 | 467 | *P. lineata* (gi:257783477) | 1.52 | 467 | *P. canaliculata* (gi:167614052) and 14 others | Successful match at 0.0% (within threshold) | Correct | Correct | Correct |
| *P. canaliculata* (GC18) | *P. canaliculata* (gi:167614052) | 0.0 | 467 | *P. lineata* (gi:257783477) | 1.52 | 467 | *P. canaliculata* (gi:167614052) and 14 others | Successful match at 0.0% (within threshold) | Correct | Correct | Correct |
| *P. canaliculata* (GC20) | *P. canaliculata* (gi:167614052) | 0.0 | 467 | *P. lineata* (gi:257783477) | 1.52 | 467 | *P. canaliculata* (gi:167614052) and 14 others | Successful match at 0.0% (within threshold) | Correct | Correct | Correct |
| *P. canaliculata* (GC7) | *P. canaliculata* (gi:167614052) | 0.0 | 467 | *P. lineata* (gi:257783477) | 1.52 | 467 | *P. canaliculata* (gi:167614052) and 14 others | Successful match at 0.0% (within threshold) | Correct | Correct | Correct |
| *P. canaliculata* (LL6) | *P. canaliculata* (gi:167614052) | 0.0 | 467 | *P. lineata* (gi:257783477) | 1.52 | 467 | *P. canaliculata* (gi:167614052) and 14 others | Successful match at 0.0% (within threshold) | Correct | Correct | Correct |
| *P. canaliculata* (gi:527138384) | *P. canaliculata* (gi:167614052) | 0.0 | 467 | *P. lineata* (gi:257783477) | 1.52 | 467 | *P. canaliculata* (gi:167614052) and 14 others | Successful match at 0.0% (within threshold) | Correct | Correct | Correct |
| *P. canaliculata* (gi:666914761) | *P. canaliculata* (gi:167614052) | 0.0 | 467 | *P. lineata* (gi:257783477) | 1.52 | 467 | *P. canaliculata* (gi:167614052) and 14 others | Successful match at 0.0% (within threshold) | Correct | Correct | Correct |
| *P. lineata* (gi:257783476) | *P. lineata* (gi:257783477) | 0.64 | 467 | *P. canaliculata* (LL2) | 1.73 | 468 | *P. lineata* (gi:257783477) | Successful match at 0.64% (within threshold) | Correct | Correct | Ambiguous |
| *P. lineata* (gi:257783477) | *P. lineata* (gi:257783476) | 0.64 | 467 | *P. canaliculata* (gi:167614052) | 1.52 | 467 | *P. lineata* (gi:257783476) | Successful match at 0.64% (within threshold) | Correct | Correct | Ambiguous |
| *P. maculata* (AN1) | *P. maculata* (AN2) | 0.0 | 467 | *P. canaliculata* (gi:167614052) | 2.18 | 467 | *P. maculata* (AN2) and 11 others | Successful match at 0.0% (within threshold) | Correct | Correct | Correct |
| *P. maculata* (AN2) | *P. maculata* (AN1) | 0.0 | 467 | *P. canaliculata* (gi:167614052) | 2.18 | 467 | *P. maculata* (AN1) and 11 others | Successful match at 0.0% (within threshold) | Correct | Correct | Correct |
| *P. maculata* (AN3) | *P. maculata* (AN1) | 0.0 | 467 | *P. canaliculata* (gi:167614052) | 2.18 | 467 | *P. maculata* (AN1) and 11 others | Successful match at 0.0% (within threshold) | Correct | Correct | Correct |
| *P. maculata* (AN4) | *P. maculata* (AN1) | 0.0 | 467 | *P. canaliculata* (gi:167614052) | 2.18 | 467 | *P. maculata* (AN1) and 11 others | Successful match at 0.0% (within threshold) | Correct | Correct | Correct |
| *P. maculata* (AN5) | *P. maculata* (CC2) | 0.0 | 467 | *P. canaliculata* (gi:167614052) | 2.41 | 467 | *P. maculata* (CC2) and 5 others | Successful match at 0.0% (within threshold) | Correct | Correct | Correct |
| *P. maculata* (CC1) | *P. maculata* (AN1) | 0.0 | 467 | *P. canaliculata* (gi:167614052) | 2.18 | 467 | *P. maculata* (AN1) and 11 others | Successful match at 0.0% (within threshold) | Correct | Correct | Correct |
| *P. maculata* (CC2) | *P. maculata* (AN5) | 0.0 | 467 | *P. canaliculata* (gi:167614052) | 2.41 | 467 | *P. maculata* (AN5) and 5 others | Successful match at 0.0% (within threshold) | Correct | Correct | Correct |
| *P. maculata* (CC3) | *P. maculata* (AN5) | 0.0 | 467 | *P. canaliculata* (gi:167614052) | 2.41 | 467 | *P. maculata* (AN5) and 5 others | Successful match at 0.0% (within threshold) | Correct | Correct | Correct |
| *P. maculata* (CC4) | *P. maculata* (AN1) | 0.0 | 467 | *P. canaliculata* (gi:167614052) | 2.18 | 467 | *P. maculata* (AN1) and 11 others | Successful match at 0.0% (within threshold) | Correct | Correct | Correct |
| *P. maculata* (CC5) | *P. maculata* (AN1) | 0.0 | 467 | *P. canaliculata* (gi:167614052) | 2.18 | 467 | *P. maculata* (AN1) and 11 others | Successful match at 0.0% (within threshold) | Correct | Correct | Correct |
| *P. maculata* (gi:1168026801) | *P. maculata* (AN1) | 0.0 | 467 | *P. canaliculata* (gi:167614052) | 2.22 | 467 | *P. maculata* (AN1) and 11 others | Successful match at 0.0% (within threshold) | Correct | Correct | Correct |
| *P. maculata* (gi:257783474) | *P. maculata* (AN1) | 1.3 | 467 | *P. canaliculata* (gi:167614052) | 2.18 | 467 | *P. maculata* (AN1) and 11 others | Successful match at 1.3% (outside threshold) | Correct | No match | No match |
| *P. maculata* (gi:257783475) | *P. maculata* (AN1) | 0.64 | 467 | *P. canaliculata* (gi:167614052) | 2.41 | 467 | *P. maculata* (AN1) and 11 others | Successful match at 0.64% (within threshold) | Correct | Correct | Correct |
| *P. maculata* (PJ2) | *P. maculata* (AN1) | 0.0 | 467 | *P. canaliculata* (gi:167614052) | 2.18 | 467 | *P. maculata* (AN1) and 11 others | Successful match at 0.0% (within threshold) | Correct | Correct | Correct |
| *P. maculata* (PJ3) | *P. maculata* (AN1) | 0.0 | 467 | *P. canaliculata* (gi:167614052) | 2.18 | 467 | *P. maculata* (AN1) and 11 others | Successful match at 0.0% (within threshold) | Correct | Correct | Correct |
| *P. maculata* (PJ4) | *P. maculata* (AN1) | 0.0 | 467 | *P. canaliculata* (gi:167614052) | 2.18 | 467 | *P. maculata* (AN1) and 11 others | Successful match at 0.0% (within threshold) | Correct | Correct | Correct |
| *P. maculata* (PJ11) | *P. maculata* (AN1) | 0.0 | 467 | *P. canaliculata* (gi:167614052) | 2.18 | 467 | *P. maculata* (AN1) and 11 others | Successful match at 0.0% (within threshold) | Correct | Correct | Correct |
| *P. maculata* (PJ15) | *P. maculata* (AN1) | 0.0 | 467 | *P. canaliculata* (gi:167614052) | 2.18 | 467 | *P. maculata* (AN1) and 11 others | Successful match at 0.0% (within threshold) | Correct | Correct | Correct |
| *P. maculata* (GC1) | *P. maculata* (AN5) | 0.0 | 467 | *P. canaliculata* (gi:167614052) | 2.41 | 467 | *P. maculata* (AN5) and 5 others | Successful match at 0.0% (within threshold) | Correct | Correct | Correct |
| *P. maculata* (GC2) | *P. maculata* (AN5) | 0.0 | 467 | *P. canaliculata* (gi:167614052) | 2.41 | 467 | *P. maculata* (AN5) and 5 others | Successful match at 0.0% (within threshold) | Correct | Correct | Correct |
| *P. maculata* (GC5) | *P. maculata* (AN5) | 0.0 | 467 | *P. canaliculata* (gi:167614052) | 2.41 | 467 | *P. maculata* (AN5) and 5 others | Successful match at 0.0% (within threshold) | Correct | Correct | Correct |
| *P. maculata* (GC11) | *P. maculata* (AN5) | 0.0 | 467 | *P. canaliculata* (gi:167614052) | 2.41 | 467 | *P. maculata* (AN5) and 5 others | Successful match at 0.0% (within threshold) | Correct | Correct | Correct |
| *P. paludosa* (gi:257783483) | *P. paludosa* (gi:257783484) | 0.22 | 465 | *P. lineata* (gi:257783477) | 4.87 | 465 | *P. paludosa* (gi:257783484 | Successful match at 0.22% (within threshold) | Correct | Correct | Correct |
| *P. paludosa* (gi:257783484) | *P. paludosa* (gi:257783483) | 0.22 | 465 | *P. lineata* (gi:257783477) | 4.88 | 465 | *P. paludosa* (gi:257783483) | Successful match at 0.22% (within threshold) | Correct | Correct | Correct |
| *P. scalaris* (gi:257783486) | *P. scalaris* (gi:257783487) | 2.61 | 470 | *P. canaliculata* (SJ2) | 7.61 | 470 | *P. scalaris* (gi:257783487) | Successful match at 2.61% (outside threshold) | Correct | No match | No match |
| *P. scalaris* (gi:257783487) | *P. scalaris* (gi:257783486) | 2.61 | 470 | *P. lineata* (gi:257783477) | 8.64 | 466 | *P. scalaris* (gi:257783486) | Successful match at 2.61% (outside threshold) | Correct | No match | No match |
